# Supplementary material for: Water in peripheral TM-interfaces of Orai1-channels triggers pore opening
Source: Commun Biol. 2024 Nov 16;7:1522. doi: 10.1038/s42003-024-07174-6 (PMC11569263; doi:10.1038/s42003-024-07174-6)
Supplement: Supplementary file 1 — Supplementary Information [file 42003_2024_7174_MOESM1_ESM.pdf]

Supplementary Information

Water in peripheral TM-interfaces of Orai1 channels triggers pore opening

Valentina Hopl[+1], Adéla Tiffner[+1,2], Armin Wutscher[1], Matthias Sallinger[1], Herwig Grabmayr[1], Magdalena Prantl[1], Maximilian Fröhlich[1], Julia Söllner[1], Sarah Weiß[1], Hadil Najjar[1], Yuliia Nazarenko[1], Selina Harant[1], Natalia Kriško[1], Marc Fahrner[1], Christina Humer[1], Carmen Höglinger[1], Heinrich Krobath[3], Daniel Bonhenry[4,*], Isabella Derler[1,*]

[1]Institute of Biophysics, JKU Life Science Center, Johannes Kepler University Linz, 4020 Linz, Austria
[2]Institute for Physiology and Pathophysiology, Johannes Kepler University Linz, Linz, 4020 Austria
[3]Institute of Theoretical Physics, Johannes Kepler University Linz, Altenbergerstraße 69, 4040 Linz, Austria
[4]Department of Physics and Materials Science, University of Luxembourg, L-1511 Luxembourg City, Luxembourg.

+ These authors contributed equally.
* Corresponding authors: daniel.bonhenry@gmail.com; isabella.derler@jku.at

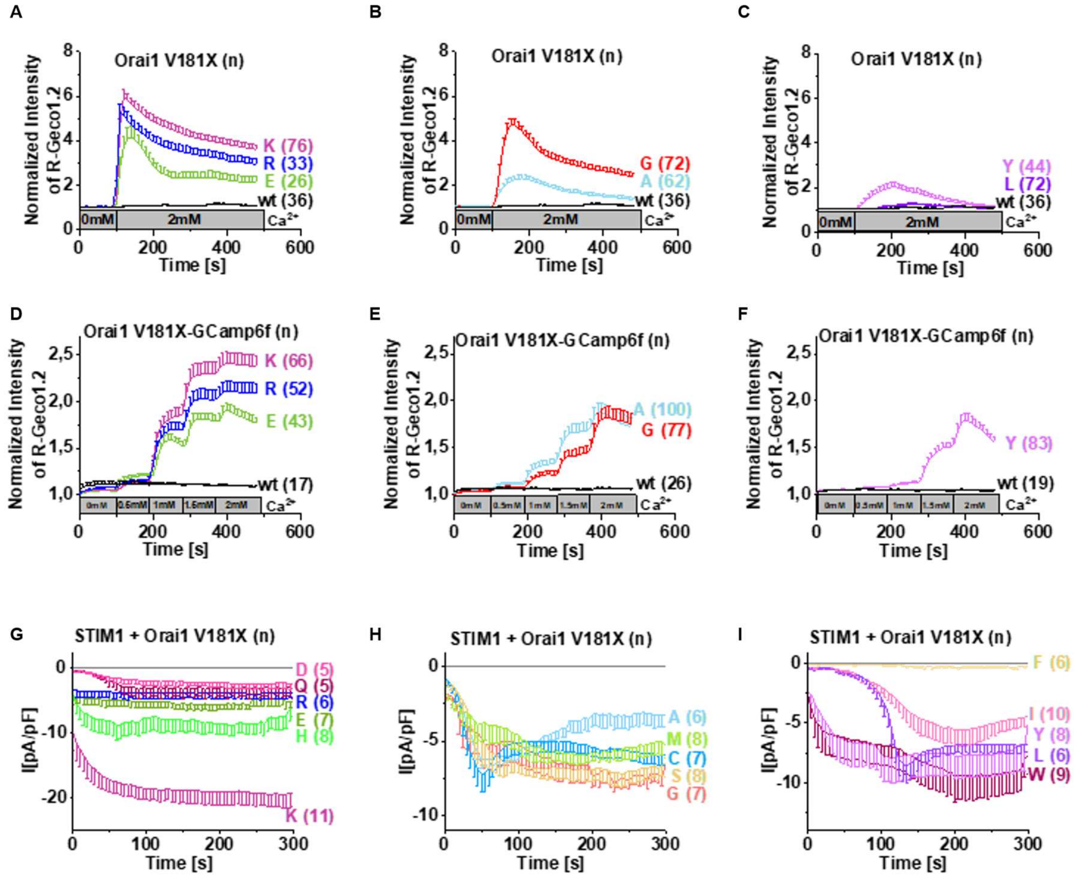

Supplementary Figure 1: Effects of V181 substitutions in Orai1 on cytosolic Ca^{2+} levels and on STIM1-mediated activation.

(A-C) Cytosolic Ca^{2+} concentrations represented by the normalized intensity of overexpressed R-Geco1.2 were monitored initially in a nominally Ca^{2+} free extracellular solution, followed by a solution containing 2 mM Ca^{2+} in HEK293 cells overexpressing Orai1-V181X (X = K/E/R (A), A/G (B), L/Y (C)) compared to Orai1. **(D-F)** Cytosolic Ca^{2+} concentrations represented by the normalized intensity of overexpressed GCamp6f were monitored initially in a nominally Ca^{2+} free extracellular solution, followed by a stepwise increase to 0,5mM, 1mM, 1,5mM and 2mM Ca^{2+} extracellularly in HEK293 cells overexpressing Orai1-V181X-GCamp6 (X = K/E/R (D), A/G (E), Y (F)). **(G–I)** Time courses of Ca^{2+} current densities after whole-cell break-in of Orai1-V181X (X=K/E/R/Q/D/H) (G), (X=G/A/C/S/M) (H), (X=F/W/Y/I/L) (I) in the presence of STIM1.

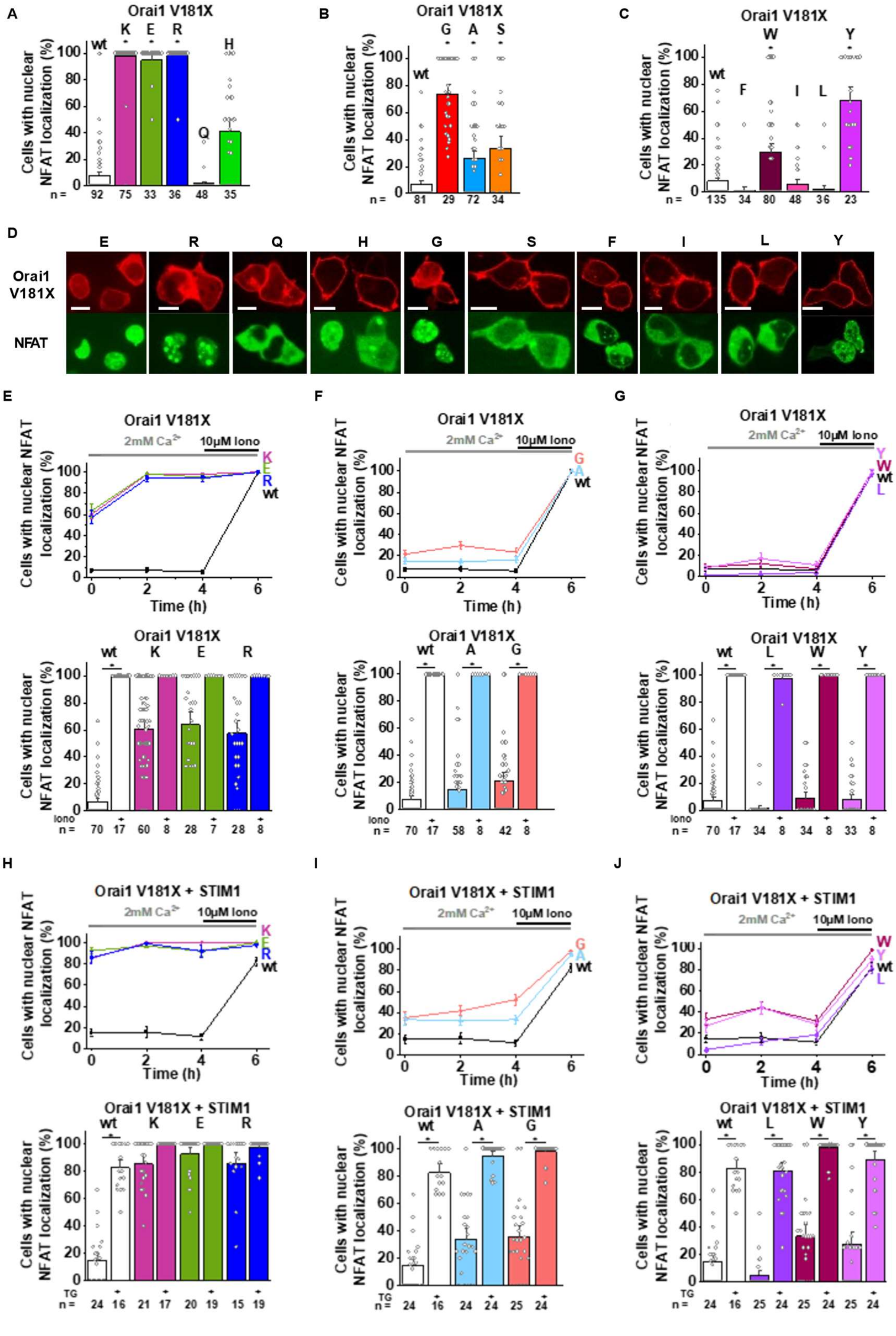
A
B
C
D
E
F
G
H
I
J
Orai1 V181X
Orai1 V181X + STIM1
Cells with nuclear NFAT localization (%)
Orai1
V181X
NFAT
2mM Ca2+
10μM Iono
Time (h)
Iono
TG
n =

Supplementary Figure 2: Effects of V181 substitutions in Orai1 on NFAT translocation.

(A–C) The average number of HEK293 cells that exhibit nuclear NFAT localization determined upon co-expression (CFP-NFAT) with Orai1 (wt) or Orai1-V181X (X=K/E/R/Q/H) (A), (X=G/A/S) (B), (X=F/W/I/L/Y) (C) mutants in the absence of STIM1 after 24 h in 2 mM Ca^{2+} containing media. For the analysis 29-119 images of cells containing in total 70-413 cells were used. **(D)** Representative images of HEK293 cells co-expressing corresponding mutants shown in (A-C) with CFP-NFAT in the presence of 2 mM Ca^{2+} after 24 h (Scale bar, 10 µm). **(E-J)** Top: Time course of NFAT translocation to the nucleus of HEK293 cells expressing Orai1-V181X (X=K/E/R) (E,H), (X=G/A) (F,I), (X=W/L/Y) (G,J) mutants in the absence of STIM1 (E-G) or the presence of STIM1 (H-J) compared to wt Orai1 after the exchange of 0mM Ca^{2+}- by 2mM Ca^{2+}-containing solution at t = 0s (E-J). After 4 hours 10µM ionomycin (iono) was perfused in cells lacking STIM1 overexpressed to rapidly increase cytosolic Ca^{2+} levels (E-G), while 2µM TG was used to induce store-depletion in STIM1-overexpressing cells (H-J). Bottom: Bar diagrams corresponding to time courses at the top of (E-J) exhibiting the average number of HEK293 cells that exhibit nuclear NFAT localization at time point t = 0 h and t = 6 h. For the presented bar diagrams, Kruskal-Wallis ANOVA was employed for statistical analyses with differences considered statistically significant at $p < 0.05$ indicated with asterisks (asterisks indicated statistical significance for wt. versus mutant; see **Supplementary Data 1**).

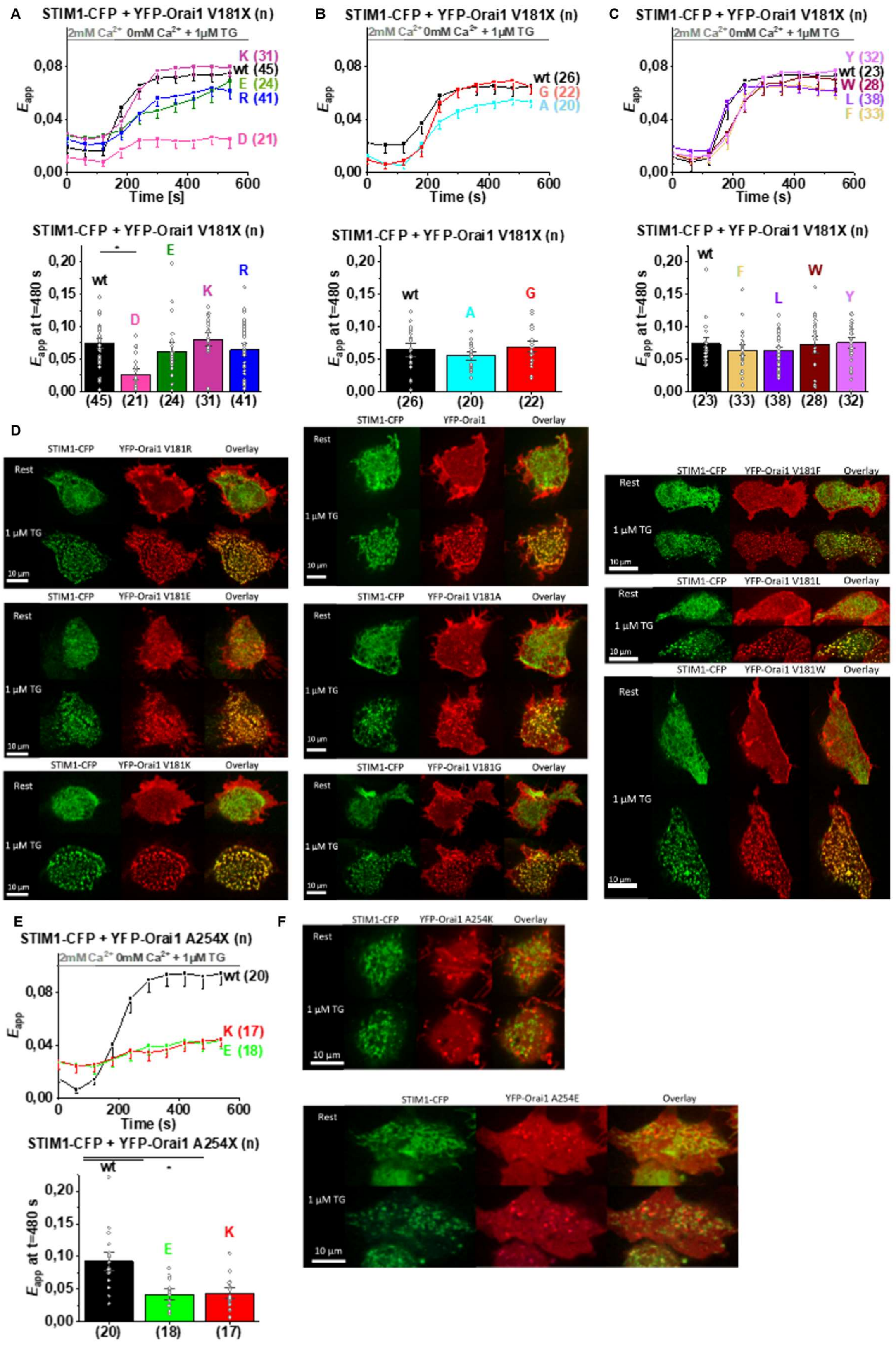
A
STIM1-CFP + YFP-Orai1 V181X (n)
2mM Ca2+ 0mM Ca2+ + 1µM TG
K (31)
wt (45)
E (24)
R (41)
D (21)
Time [s]
B
STIM1-CFP + YFP-Orai1 V181X (n)
wt (26)
G (22)
A (20)
Time (s)
C
STIM1-CFP + YFP-Orai1 V181X (n)
Y (32)
wt (23)
W (28)
L (38)
F (33)
Time (s)
Eapp at t=480 s
(45) (21) (24) (31) (41)
(26) (20) (22)
(23) (33) (38) (28) (32)
D
STIM1-CFP
YFP-Orai1 V181R
YFP-Orai1
YFP-Orai1 V181F
YFP-Orai1 V181E
YFP-Orai1 V181A
YFP-Orai1 V181L
YFP-Orai1 V181K
YFP-Orai1 V181G
YFP-Orai1 V181W
Overlay
Rest
1 µM TG
10 µm
E
STIM1-CFP + YFP-Orai1 A254X (n)
wt (20)
K (17)
E (18)
(20) (18) (17)
F
YFP-Orai1 A254K
YFP-Orai1 A254E

Supplementary Figure 3: Effects of Orai1-V181X mutants on coupling to STIM1. A)-C) Top: Time courses of FRET (E_{app}) monitoring the interaction of STIM1 with Orai1-V181X (X=K/E/R/D) (A), (X=G/A) (B), (X= L/F/Y/W) (C) mutants compared to wt Orai1 when switching from a 2mM Ca^{2+}-containing solution to a 0mM Ca^{2+}/1 μM thapsigargin (TG) solution inducing STIM1/Orai1 interaction. Bottom: Bar diagrams corresponding to time courses at the top of (A-C) exhibiting FRET (E_{app}) at time point t = 480 s. **D)** Confocal fluorescence microscopy images of representative cells before and after treatment with 1μM TG showing STIM1-CFP, YFP-Orai1 or YFP-Orai1-V181X (X=R,E,K,A,G,F,L,W). **E)** Top: Time courses of FRET (E_{app}) monitoring the interaction of STIM1 with Orai1-A254X (X=K/E) mutants compared to wt Orai1 when switching from a 2mM Ca^{2+}-containing solution to a 0mM Ca^{2+}/1 μM thapsigargin (TG) solution inducing STIM1/Orai1 interaction. Bottom: Bar diagrams corresponding to time courses at the top of (E) exhibiting FRET (E_{app}) at time point t = 480 s. **F)** Confocal fluorescence microscopy images of representative cells before and after treatment with 1 μM TG showing STIM1-CFP, YFP-Orai1or YFP-Orai1-A254X (X=K,E).

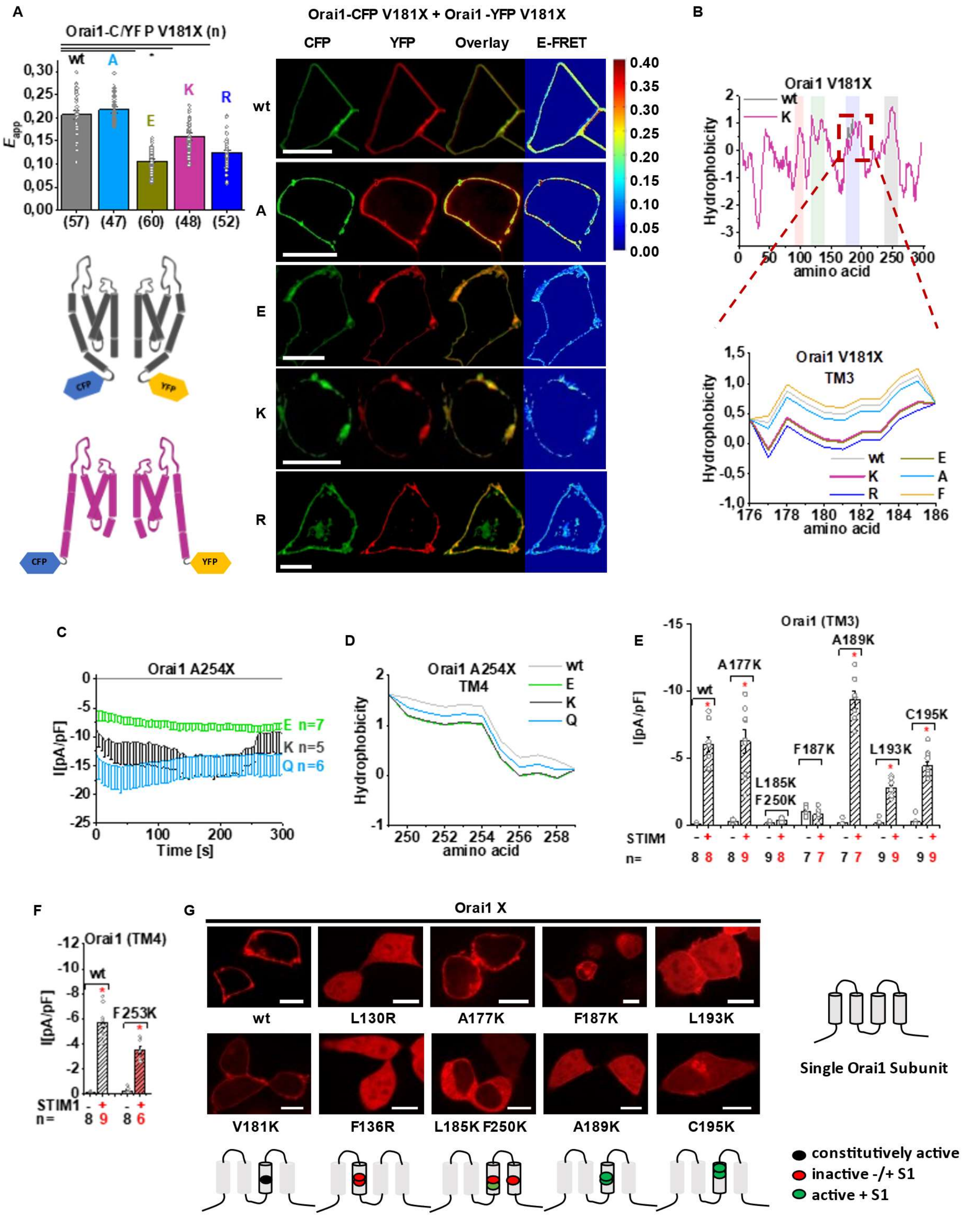

Supplementary Figure 4: Effects of certain substitutions by charged residues on FRET of YFP-/CFP-labeled Orai1 mutants, on overall hydrophobicity of the TM containing the substitution, on PM localization and current activation.

(A) Bar diagram depicting FRET of fluorescently labeled Orai1-V181X-CFP/-YFP mutants (X = A,E,K,R) in comparison with Orai1-CFP/-YFP (wt). Image series depict Orai1-CFP/-YFP variants, overlay and pixelwise calculated E_{app} index for a representative cell (Scale bar, 10 µm). Schemes represent the

potential interpretation based on detected outcome, namely a reduction in FRET observed for the constitutive Orai1-V181X (X=K;R;E) mutants, which potentially indicates a conformational change. **(B)** Hydrophobicity profiles of the full-length Orai1 and Orai1-V181K based on the Roseman[1] hydrophobicity scale using the Expasy-Protscale prediction program. Inlet represents the hydrophobicity plots of TM3 region (amino acid 176–186) and of Orai1-V181X (X=K/R/E/A/F) mutants compared to wt Orai1. **(C)** Time courses of Ca^{2+} current densities after whole-cell break-in of Orai1-A254X (X=K/E/Q) in the presence of STIM1. **(D)** Hydrophobicity plots of the Orai1 TM4 region (amino acid 248–260) for Orai1-A254X (X=K/E/Q) mutants compared to wt Orai1. **(E-F)** Block diagrams of maximal whole-cell current densities detected for Orai1-A177K, Orai1-L185K-F250K, Orai1-F187K, Orai1-A189K, Orai1-L193K, Orai1-C195K (E), and Orai1-F253K (F) compared to Orai1 (wt) in the absence and presence of STIM1. **(G)** Representative images of Orai1 TM2 (Orai1-L130R, Orai1-F136R) and TM3 (Orai1-A177K, Orai1-L185K-F250K, Orai1-F187K, Orai1-A189K, Orai1-L193K, Orai1-C195K) mutants that reveal partial or complete loss of PM localization upon substitution to charged residues compared to wt Orai1 and Orai1-V181K. For the presented bar diagram, Welch-ANOVA was employed for statistical analyses with differences considered statistically significant at $p < 0.05$ indicated with asterisks (asterisks indicate statistical significance of Orai1 mutant currents without compared to with STIM1; see **Supplementary Data 1**).

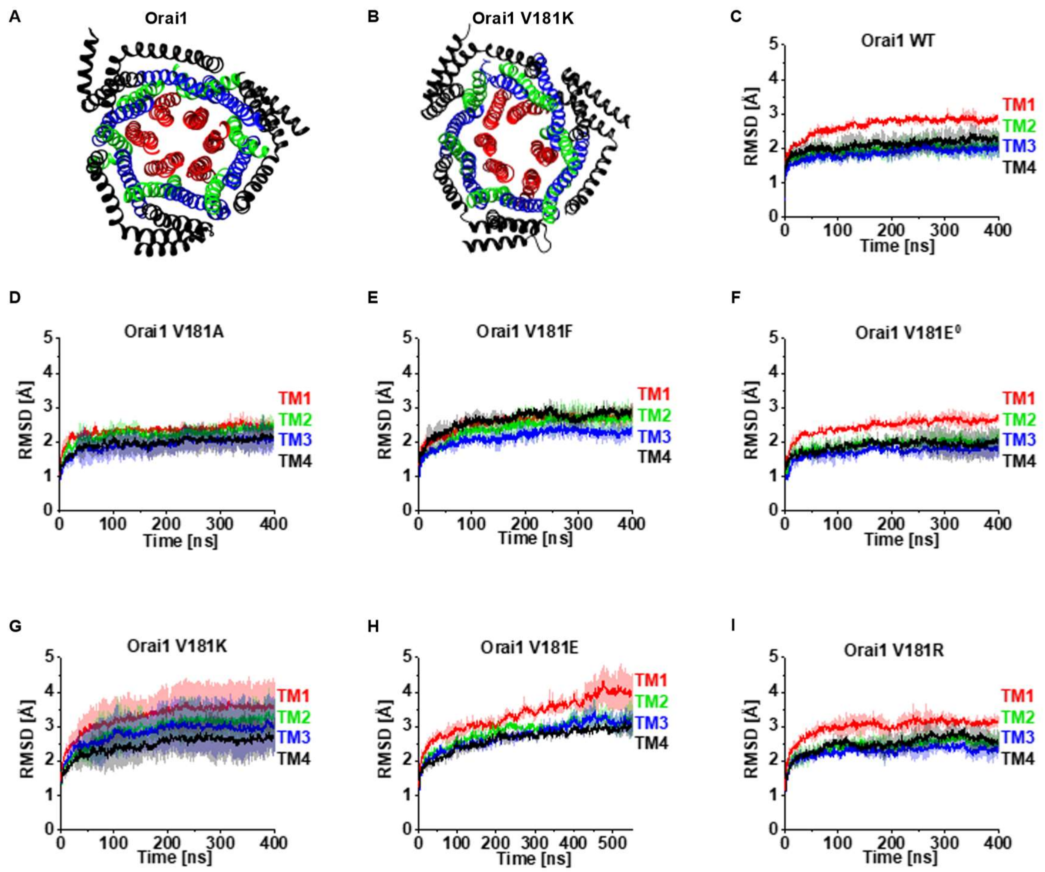

Supplementary Figure 5: Structural stability for the wt Orai1 and its mutants.

(A-B) Schemes depict top view of Orai1 (A) and Orai1-V181K (B) structures obtained from MD simulations. **(C-I)** Increase of the RMSD calculated for the backbone of the wt Orai1 (C), Orai1-V181A (D), Orai1-V181F (E), Orai1-V181E^0 (F), Orai1-V181K (G), Orai1-V181E (H) and Orai1-V181R (I) mutants as a function of the simulation time. The RMSD for the TM1 (residue 80 to 110), TM2 (residue 120 to 140), TM3 (residue 174 to 194) and TM4 (residue 235 to 255) are shown in red, green, blue and black, respectively. The shaded areas correspond to the statistical error over the three replicas.

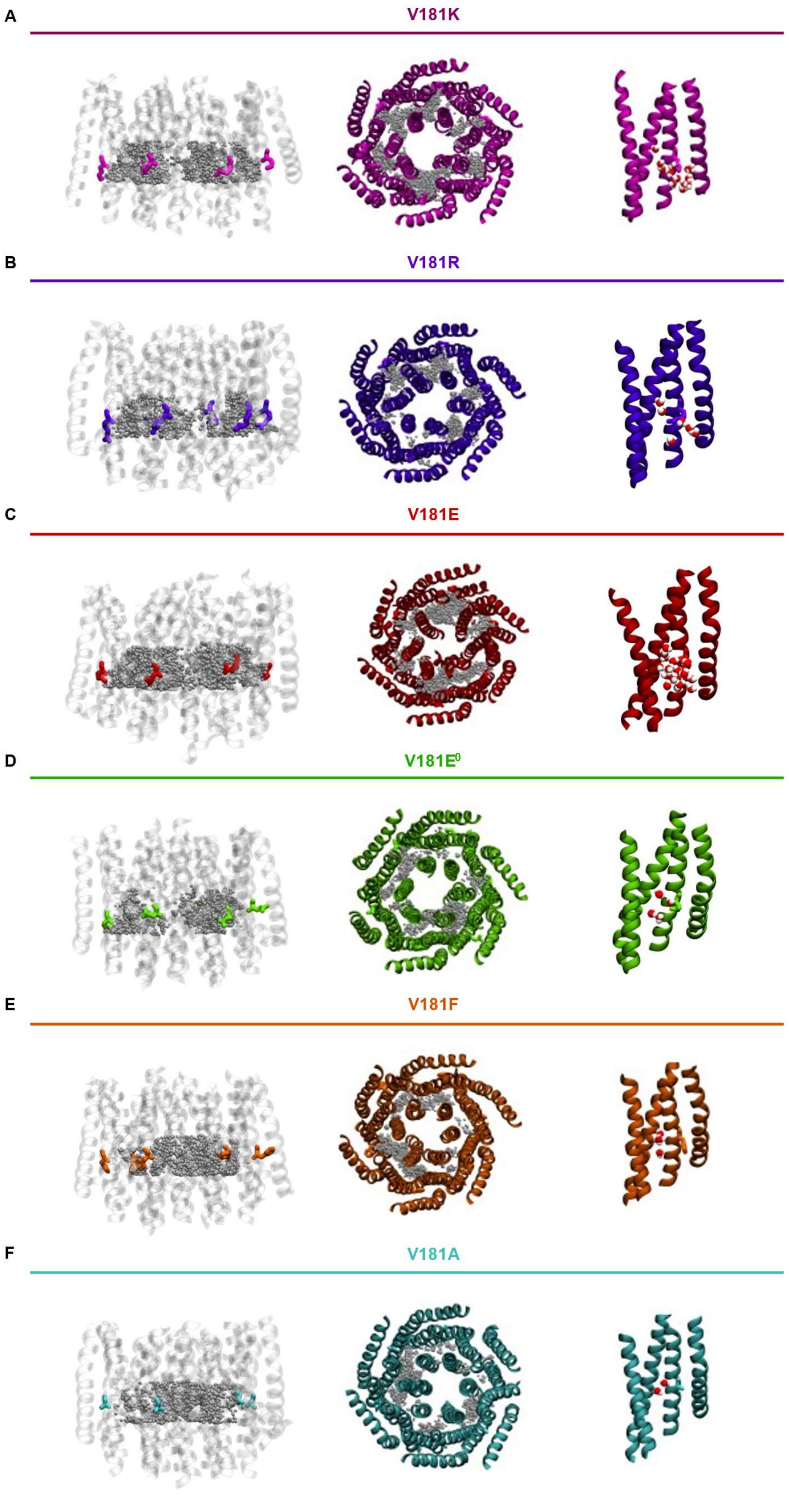
A
V181K
B
V181R
C
V181E
D
V181E^{0}
E
V181F
F
V181A

Supplementary Figure 6: Hydration around V181 in TM3 in different Orai1-V181X (X=K,R,E,E^0,F,A) mutants. A) – F) Left: Side view of representative snapshots for Orai1-V181K (A), Orai1-V181R (B), Orai1-V181E (C), Orai1-V181E^0 (D), Orai1-V181F (E) and Orai1-V181A (F) depicted as grey ribbon using a transparent representation with residue 181 shown as sticks in purple, blue, red, green, orange and cyan, respectively. The positions occupied by oxygen atoms were selected around 6 Å from the Cα from residue 181 and 6 Å above and below from the z position of residue 181. Each grey bead represents an oxygen atom from the water molecules from a superposition of 25 snapshots covering 50 ns between 350 to 400 ns simulations. Middle: Bottom view of representative snapshots from the channel displayed as ribbons with the color chosen to match the color code for the mutants. Right: Representative snapshots of a subunit and the water molecules around 6 Å from residue 181. Water molecules around the residues are depicted using red balls for the oxygen atoms and white balls for the hydrogen atoms.

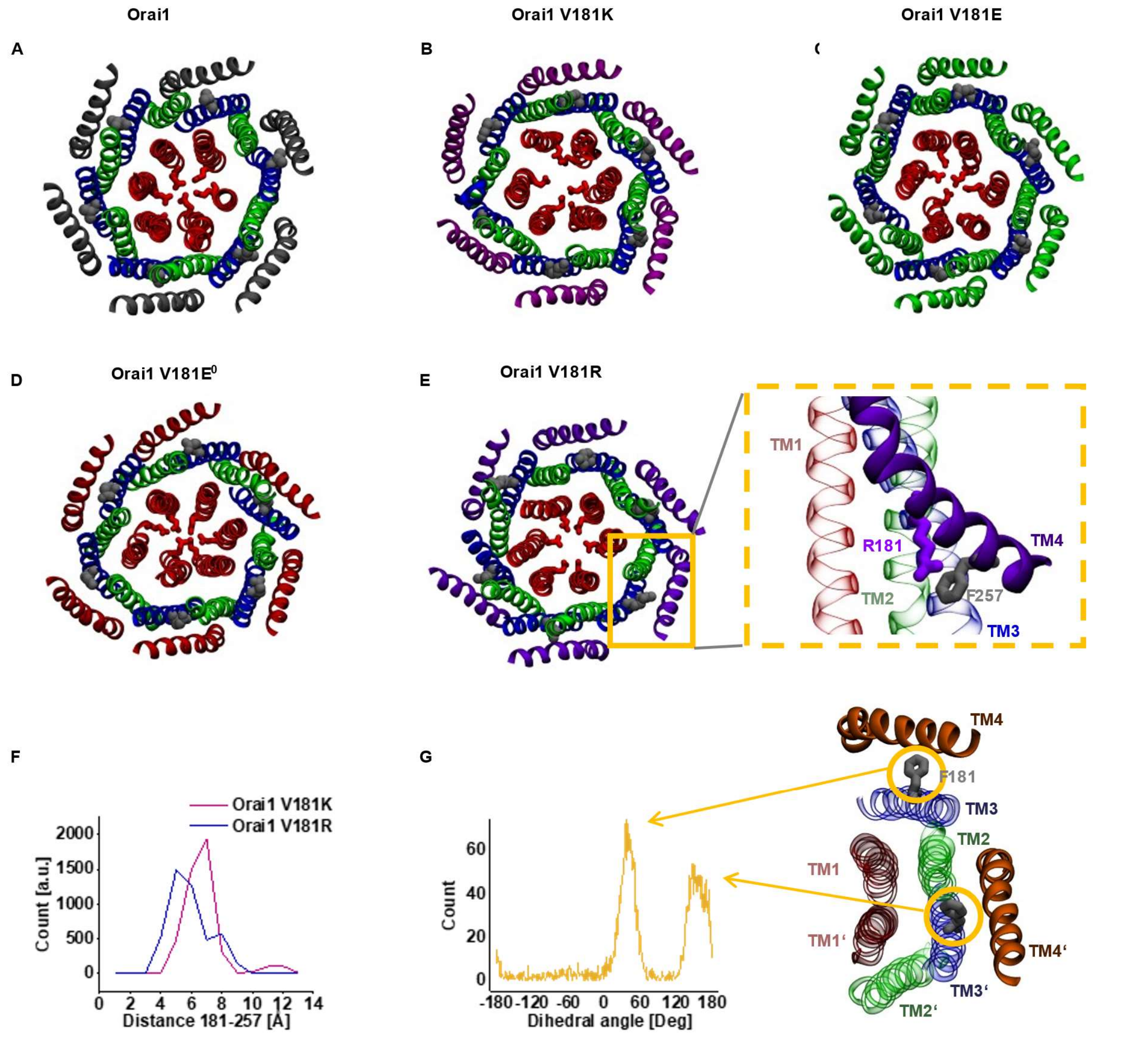

Supplementary Figure 7: Orientation of substituted residues at position V181 and their interplay with other residues.

(A-E) Representative snapshots of the transmembrane domain of the protein at the end of a 250 ns long MD simulation. TM1 (residues 80 to 110), TM2 (residues 120 to 150) and TM3 (residues 170 to 195) are depicted as red, green and blue transparent ribbons, respectively. The TM4 helix is shown using solid ribbons with the following color code applied to the wt Orai1 (black) (A) and its mutants V181K (purple) (B), V181E (dark red) (C), V181E^0 (red) (D) and V181R (blue) (E). **(E orange inlet)** Magnification on a subunit of a representative configuration of R181 (blue stick) in interaction with F257 (silver stick) which prevents the arginine residue from projecting itself toward the conducting pore. **(F)** Distribution of the distances between the center of geometry of the heavy atoms in the guanidinium moiety from R181 and the center of geometry of the heavy atoms in the phenyl ring from F257(solid purple line) and distances between the center of geometry of the heavy atoms of the amino moiety (nitrogen atom) from K181 and the center of geometry of the heavy atoms in the phenyl ring from F257(solid purple line). The distances used to plot the distributions were calculated from the last 50 ns of a 400 ns long simulations in all subunits from the three replicas for each mutants. **(G)** Left: Distribution of the dihedral angles during the last 50 ns showing the two different configurations

adopted by F181. Wedged between TM4 and TM3 for the peak around 45 degrees and TM3 and TM2 between 145 degrees. Right: Representative snapshots of the two configurations adopted by F181 seen during the simulation. TM1 (residues 80 to 110), TM2 (residues 120 to 150) and TM3 (residues 170 to 195) are depicted as red, green and blue transparent ribbons respectively. The TM4 helix is shown using orange solid ribbons. F181 is depicted using grey sticks representation.

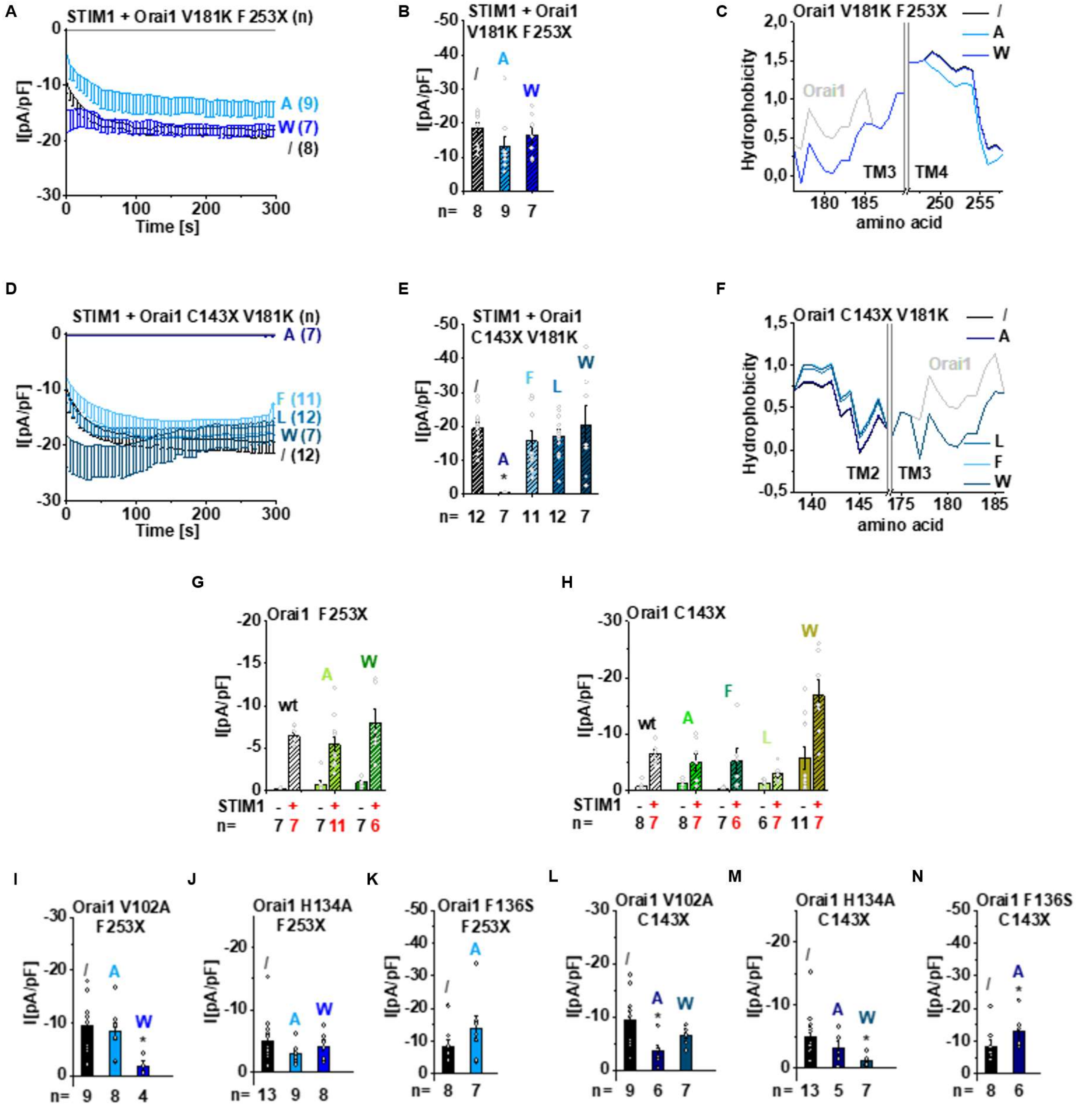

Supplementary Figure 8: Effects of hydrophobic substitutions on Orai1-V181K when co-expressed with STIM1, on overall hydrophobicity along TM3 and TM4 and on wt Orai1 and on other Orai1 GoF mutants.

(A, D) Time courses of Ca^{2+} current densities after whole-cell break-in of Orai1-V181K-F253A/W (A) and Orai1-C143A/F/L/W-V181K (D) compared to Orai1-V181K in the presence of STIM1. **(B, E)** Block diagrams of maximal whole-cell current densities of mutants recorded in (A, D) compared to Orai1-V181K (indicated by /) in the presence of STIM1. **(C, F)** Plots represent the hydrophobic profile of the respective TM domains covering the range of amino acids according to the position of the exchanged residues. For Orai1-V181K-F253A/W (C), TM3 (aa:176-190) and TM4 (aa: 246-258) regions are shown. For Orai1-C143A/F/L/W-V181K (F), TM2 (aa:138-148) and TM3 (aa:174-186) regions are shown. Different TM domains are distinguished by the lines indicating a break within the X-axis. **(G-H)** Block diagrams of maximal whole-cell current densities of single point mutants Orai1-F253A/W (G) and Orai1-C143A/F/L/W (H) compared to wt Orai1 in the absence and presence of STIM1. **(I-N)** Bar diagrams of maximal whole-cell current densities of single (Orai1-V102A (I,L), Orai1-H134A (J,M),

Orai1-F136S (K,N); (indicated by /)) and corresponding double mutants (Orai1-V102A-F253X (X=A,W), Orai1-H134A-F253X (X=A,W), Orai1-F136S-F253X (X=A), Orai1-V102A-C143X (X=A,W), Orai1-H134A-C143X (X=A,W), Orai1-F136S-C143X (X=A)) in the absence of STIM1. For the presented bar diagrams, ANOVA (one-way/Kruskal-Wallis ANOVA) or Welch-ANOVA was employed for statistical analyses with differences considered statistically significant at $p < 0.05$ indicated with asterisks (asterisks indicate significant difference between wt and mutant (B,E) or between single and double mutant (I-N); see **Supplementary Data 1**).

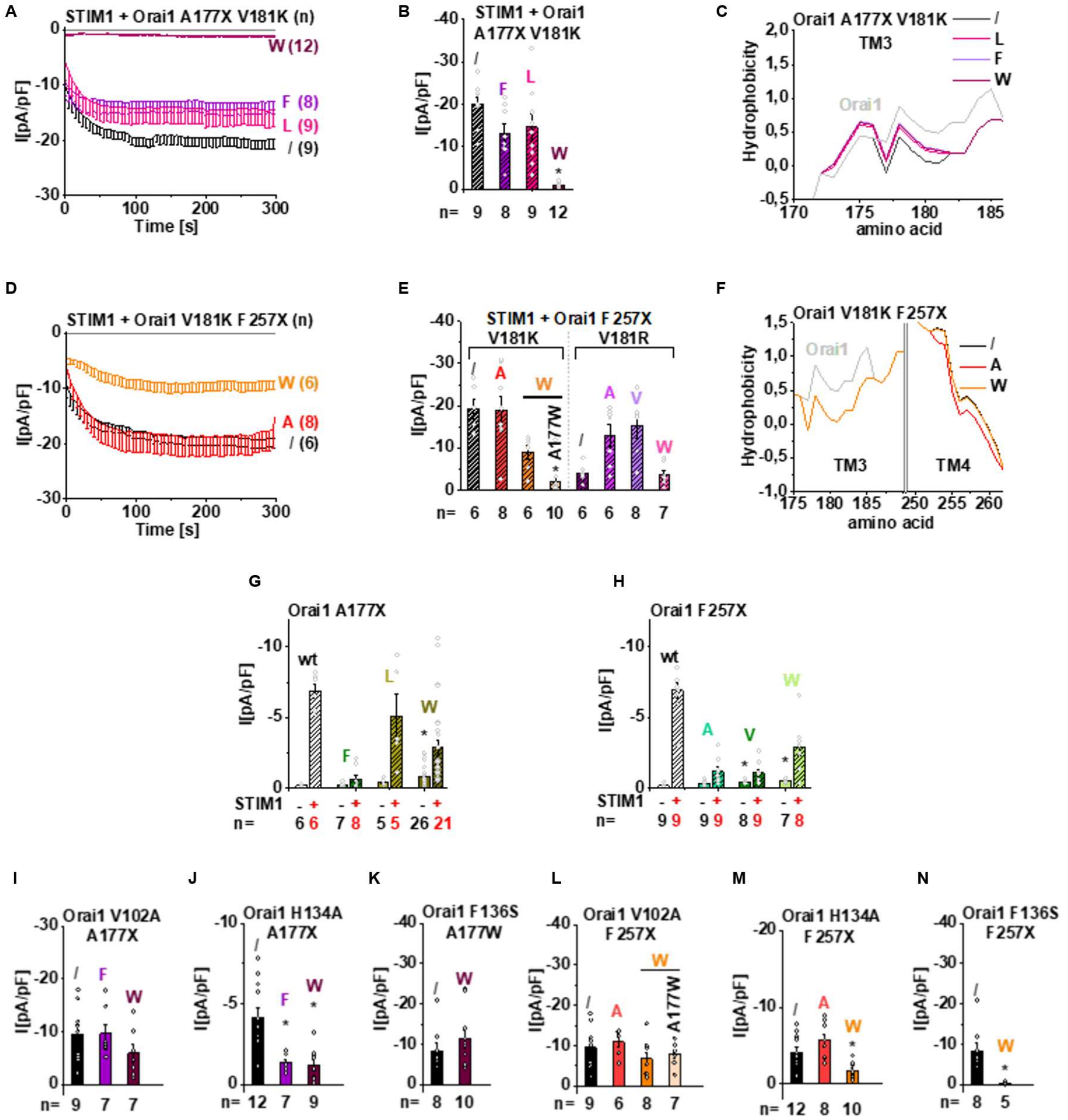

Supplementary Figure 9: Effects of hydrophobic substitutions on Orai1-V181K when co-expressed with STIM1 and on overall hydrophobicity along TM3 and TM4 and on wt Orai1 and on other Orai1 GoF mutants.

(A, D) Time courses of Ca^{2+} current densities after whole-cell break-in of Orai1-A177F/L/W-V181K (A) and Orai1-V181K-F257A/W (D) in the presence of STIM1. **(B, E)** Block diagrams of maximal whole-cell current densities of mutants recorded in (A, D) compared to Orai1-V181K (indicated by /) in the presence of STIM1. Block diagram (E) additionally shows the whole-cell current densities of Orai1-A177W-V181K-F257W and Orai1-V181R-F257A/V/W in the presence of STIM1. **(C, F)** Plots represent the hydrophobic profile of the respective TM domains covering the range of amino acids according to the position of the exchanged residues. For Orai1-A177F/L/W-V181K (C), TM3 (aa:170-186) is shown. For Orai1-V181K-F257A/W (F), TM3 (aa:174-186) and TM4 (aa:249-262) regions are shown. Different TM domains are distinguished by the lines indicating a break within the X-axis. **(G-H)** Block diagrams of maximal whole-cell current densities detected for single mutants Orai1-A177F/L/W (G) and Orai1-

F257A/V/W (H) compared to wt Orai1 in the absence and presence of STIM1. **(I-N)** Bar diagrams of maximal whole-cell current densities of single (Orai1-V102A (I,L), Orai1-H134A (J,M), Orai1-F136S (K,N); (indicated by /)) and corresponding double mutants (Orai1-V102A-A177X (X=F,W), Orai1-H134A-A177X (X=F,W), Orai1-F136S-A177X (X=W), Orai1-V102A-F257X (X=A,W), Orai1-H134A-F257X (X=A,W), Orai1-F136S-F257X (X=W)) in the absence of STIM1. For the presented bar diagrams, ANOVA (one-way/Kruskal-Wallis ANOVA) or Welch-ANOVA was employed for statistical analyses with differences considered statistically significant at $p < 0.05$ indicated with asterisks (asterisks indicate significant difference between wt and mutant without STIM1 (B,E,G,H) or between single and double mutant (I-N); see **Supplementary Data 1**).

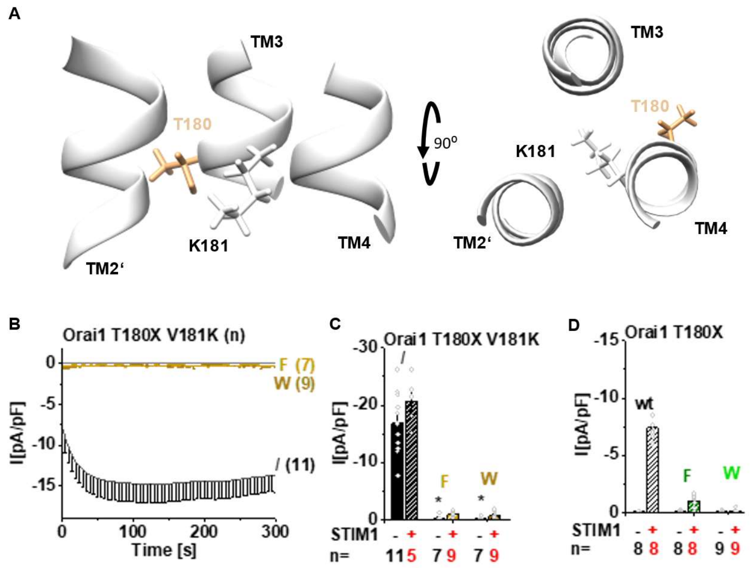

Supplementary Figure 10: Effects of hydrophobic substitutions of T180 on Orai1-V181K and Orai1. **(A)** Schematics showing side and top views of the TM3/TM4-interface along with TM2' of the next subunit with the tested residues K181, T180. **(B)** Time courses of Ca^{2+} current densities after whole-cell break-in of Orai1-T180F/W-V181K compared to Orai1-V181K in the absence of STIM1. **(C)** Block diagram of maximal whole-cell current densities of mutants recorded in (B) in the absence and presence of STIM1 (Orai1-V181K is indicated by /). **(D)** Block diagram of maximal whole-cell current densities detected for single mutant Orai1-T180F/W compared to wt Orai1 in the absence and presence of STIM1. For the presented bar diagrams, ANOVA (one-way/Kruskal-Wallis ANOVA) or Welch-ANOVA was employed for statistical analyses with differences considered statistically significant at $p < 0.05$ indicated with asterisks (see **Supplementary Data 1**).

Supplementary Table 1: Comparison of interactions involving water around Orai1-V181.

We tabulate the optimal stabilising electrostatic potential energy *E* for water-water (permanent dipole-dipole) interactions, water-charge (permanent dipole - monopole) interactions and water-non-polar (permanent dipole-induced dipole) interactions around Orai1-V181. The valine side-chain has been modeled as a united atom with radius 1.67 Å as computed by VMD [2]. Water molecules have been modeled as spheres with radius 1.4 Å[3]. Experimental and theoretical water dipole estimates and TIP3P water dipole moments were used to resemble the respective scenario[4–6]. A weighted average of three carbon and seven hydrogen atom polarizabilities taken from[7] has been used to compute the water-non polar interactions. Potential energies are given in units of k_BT at T = 293 K.

***E* in k_BT**	**TIP3P water**	**water dipole estimate**
water-water interaction	-11.88	-18.89 (in bulk water)
water-point charge interaction	-28.91	-36.45 (in bulk water) || -22.98 (in protein)
water-non-polar interaction	-0.155	-0.09 (in protein)

Both in experiment and in simulations, water-point charge interactions provide more electrostatic stabilization than water-water or water-non-polar interactions indicating the pertinent existence of a water hydration shell around charged V181X Orai1 mutants. The interactions have been computed using reference[8] and are listed below.

water-water interaction

$$V(r) = \frac{-p_1 p_2}{4\pi\epsilon_0 r^3}[2cos(\theta_1)cos(\theta_2) - sin(\theta_1)sin(\theta_2)cos(\phi)]$$

with an optimal stabilization, if the dipoles are aligned in line

$$V(r) = \frac{-2p_1 p_2}{4\pi\epsilon_0 r^3}$$

water-point charge interaction

$$V(r) = \frac{-qp}{4\pi\epsilon_0 r^2}cos(\theta)$$

with an optimal stabilization, if Θ = 2kπ with an integer k.

$$V(r) = \frac{-qp}{4\pi\epsilon_0 r^2}$$

water-non-polar interaction

$$V(r) = \frac{-p^2\alpha}{4\pi\epsilon_0 r^6}$$

water dipoles are taken to rotate freely near non-polar molecules

Throughout the table above, *p* denotes a dipole moment, *q* denotes a charge in their respective SI units and α denote atomic polarizabilities in units of m^3. *r* is the distance between the mid-point(s) along the two charges constituting the dipole(s) or the distance between this dipole center and the point charge. In all cases, these definitions co-incide with the sphere center of our approximation. The following dipole moments have been used: *p*(TIP3P) = 2.3 D[6], *p*(water, bulk) = 2.9 D[5], *p*(water, protein) = 1.83 D[4] with 1 D = $3.336.10^{-30}$ Cm.

REFERENCES:

1. Roseman, M. A. Hydrophilicity of polar amino acid side-chains is markedly reduced by flanking peptide bonds. *J Mol Biol* **200**, 513–522 (1988).

2. Humphrey, W., Dalke, A. & Schulten, K. VMD: visual molecular dynamics. *J Mol Graph* **14**, 27-28,33-38 (1996).

3. Camisasca, G., Pathak, H., Wikfeldt, K. T. & Pettersson, L. G. M. Radial distribution functions of water: Models vs experiments. *J Chem Phys* **151**, (2019).

4. Morozenko, A., Leontyev, I. V. & Stuchebrukhov, A. A. Dipole Moment and Binding Energy of Water in Proteins from Crystallographic Analysis. *J Chem Theory Comput* **10**, 4618–4623 (2014).

5. Badyal, Y. S. *et al.* Electron distribution in water. *J Chem Phys* **112**, 9206–9208 (2000).

6. Mark, P. & Nilsson, L. Structure and Dynamics of the TIP3P, SPC, and SPC/E Water Models at 298 K. *J Phys Chem A* **105**, 9954–9960 (2001).

7. Miller, T. M. & Bederson, B. Atomic and Molecular Polarizabilities-A Review of Recent Advances. in 1–55 (1978). doi:10.1016/S0065-2199(08)60054-8.

8. Israelachvili, J. N. *Intermolecular and Surface Forces*. (Elsevier, 2011). doi:10.1016/C2009-0-21560-1.

Supplementary Table 2: List of primers

Nr.	Figure	POI	Mutant	Template	forward primer seq. (5'-> 3') / reverse primer seq. (5'-> 3')
1	1B	Orai1	V181Q	pEYFP YFP-Orai1	TGGGCCTTCTCCACCCAGATCGGCACGCTGCTC<br>GAGCAGCGTGCCGATCTGGGTGGAGAAGGCCCA
2	1B	Orai1	V181D	pEYFP YFP-Orai1	GCCTTCTCCACCGACATCGGCACGCTG<br>AGCGTGCCGATGTCGGTGGAGAAGGC
3	1B	Orai1	V181H	pEYFP YFP-Orai1	GGCCTTCTCCACCCACATCGGCACGCTG<br>CAGCGTGCCGATGTGGGTGGAGAAGGCC
4	1B	Orai1	V181E	pEYFP YFP-Orai1	GCCTTCTCCACCGAGATCGGCACGCTGC<br>GCAGCGTGCCGATCTCGGTGGAGAAGGC
5	1B	Orai1	V181R	pEYFP YFP-Orai1	GGCCTTCTCCACCCGCATCGGCACGCTG<br>CAGCGTGCCGATGCGGGTGGAGAAGGCC
6	1B	Orai1	V181K	pEYFP YFP-Orai1	TGGGCCTTCTCCACCAAGATCGGCACGCTGCTC<br>GAGCAGCGTGCCGATCTTGGTGGAGAAGGCCCA
7	1C	Orai1	V181C	pEYFP YFP-Orai1	GGGCCTTCTCCACCTGCATCGGCACGCTGC<br>GCAGCGTGCCGATGCAGGTGGAGAAGGCCC
8	1C	Orai1	V181M	pEYFP YFP-Orai1	GGCCTTCTCCACCATGATCGGCACGCTGC<br>GCAGCGTGCCGATCATGGTGGAGAAGGCC
9	1C	Orai1	V181S	pEYFP YFP-Orai1	GGGCCTTCTCCACCAGCATCGGCACGCTGC<br>GCAGCGTGCCGATGCTGGTGGAGAAGGCCC
10	1C	Orai1	V181G	pEYFP YFP-Orai1	GCCTTCTCCACCGGCATCGGCACGCTG<br>CAGCGTGCCGATGCCGGTGGAGAAGGC
11	1C	Orai1	V181A	pEYFP YFP-Orai1	GCCTTCTCCACCGCCATCGGCACGCTG<br>CAGCGTGCCGATGGCGGTGGAGAAGGC
12	1D	Orai1	V181L	pEYFP YFP-Orai1	GCCTTCTCCACCCTCATCGGCACGC<br>GCGTGCCGATGAGGGTGGAGAAGGC
13	1D	Orai1	V181F	pEYFP YFP-Orai1	GGCCTTCTCCACCTTCATCGGCACGCT<br>AGCGTGCCGATGAAGGTGGAGAAGGCC
14	1D	Orai1	V181I	pEYFP YFP-Orai1	GGCCTTCTCCACCATCATCGGCACGCT<br>GGCCTTCTCCACCATCATCGGCACGCT
15	1D	Orai1	V181Y	pEYFP YFP-Orai1	CTGGGCCTTCTCCACCTATATCGGCACGCTGCTCT<br>AGAGCAGCGTGCCGATATAGGTGGAGAAGGCCCAG
16	1D	Orai1	V181W	pEYFP YFP-Orai1	TGGGCCTTCTCCACCTGGATCGGCACGCTGCTC<br>GAGCAGCGTGCCGATCCAGGTGGAGAAGGCCCA
17	2B	Orai1	A254K	pEYFP YFP-Orai1	GCCTGATCTTTATCGTCTTCAAGGTCCACTTCTACCGCTCACT<br>AGTGAGCGGTAGAAGTGGACCTTGAAGACGATAAAGATCAGGC
18	2B	Orai1	A254E	pEYFP YFP-Orai1	CTGATCTTTATCGTCTTCGAGGTCCACTTCTACCGCTCAC

					GTGAGCGGTAGAAGTGGACCTCGAAGACGATAAAGATCAG
19	2B	Orai1	A254Q	pEYFP YFP-Orai1	CCTGATCTTTATCGTCTTCCAGGTCCACTTCTACCGCTCAC
					GTGAGCGGTAGAAGTGGACCTGGAAGACGATAAAGATCAGG
20	S4E	Orai1	A177K	pEYFP YFP-Orai1	CATCGAGCTGGCCTGGAAGTTCTCCACCGTCATCG
					CGATGACGGTGGAGAACTTCCAGGCCAGCTCGATG
21	S4E	Orai1	L185K	pEYFP YFP-Orai1	CACCGTCATCGGCACGAAGCTCTTCCTAGCTGAG
					CTCAGCTAGGAAGAGCTTCGTGCCGATGACGGTG
22	S4E	Orai1	F250K	pEYFP YFP-Orai1	GCCCTTCGGCCTGATCAAGATCGTCTTCGCCGTCC
					GGACGGCGAAGACGATCTTGATCAGGCCGAAGGGC
23	S4E	Orai1	F187K	pEYFP YFP-Orai1	CATCGGCACGCTGCTCAAGCTAGCTGAGGTGGTGC
					GCACCACCTCAGCTAGCTTGAGCAGCGTGCCGATG
24	S4E	Orai1	A189K	pEYFP YFP-Orai1	ATCGGCACGCTGCTCTTCCTAAAGGAGGTGGTGCTGC
					GCAGCACCACCTCCTTTAGGAAGAGCAGCGTGCCGAT
25	S4E	Orai1	L193K	pEYFP YFP-Orai1	CCTAGCTGAGGTGGTGAAGCTCTGCTGGGTCAAG
					CTTGACCCAGCAGAGCTTCACCACCTCAGCTAGG
26	S4E	Orai1	C195K	pEYFP YFP-Orai1	CTGAGGTGGTGCTGCTCAAGTGGGTCAAGTTCTTGCC
					GGCAAGAACTTGACCCACTTGAGCAGCACCACCTCAG
27	S4F	Orai1	F253K	pEYFP YFP-Orai1	GGCCTGATCTTTATCGTCAAGGCCGTCCACTTCTACCGC
					GCGGTAGAAGTGGACGGCCTTGACGATAAAGATCAGGCC
28	S4G	Orai1	L130R	pEYFP YFP-Orai1	TGCACCACAGTGCGGGTGGCTGTGCAC
					GTGCACAGCCACCCGCACTGTGGTGCA
29	S4G	Orai1	F136R	pEYFP YFP-Orai1	GGTGGCTGTGCACCTGCGTGCGCTCATGATCAGC
					GCTGATCATGAGCGCACGCAGGTGCACAGCCACC
30	6B	Orai1	F253A	pEYFP YFP-Orai1	GCCTGATCTTTATCGTCGCCGCCGTCCACTTCTACC
					GGTAGAAGTGGACGGCGGCGACGATAAAGATCAGGC
31	6B	Orai1	F253W	pEYFP YFP-Orai1	CTTCGGCCTGATCTTTATCGTCTGGGCCGTCCACTTC
					GAAGTGGACGGCCCAGACGATAAAGATCAGGCCGAAG
32	6D	Orai1	C143A	pEYFP YFP-Orai1	GCTCATGATCAGCACCGCCATCCTGCCCAACATC
					GATGTTGGGCAGGATGGCGGTGCTGATCATGAGC
33	6D	Orai1	C143F	pEYFP YFP-Orai1	CTCATGATCAGCACCTTCATCCTGCCCAACATC
					GATGTTGGGCAGGATGAAGGTGCTGATCATGAG
34	6D	Orai1	C143L	pEYFP YFP-Orai1	CGCTCATGATCAGCACCTTAATCCTGCCCAACATCGAG
					CTCGATGTTGGGCAGGATTAAGGTGCTGATCATGAGCG
35	6D	Orai1	C143W	pEYFP YFP-Orai1	CATGATCAGCACCTGGATCCTGCCCAACATC
					GATGTTGGGCAGGATCCAGGTGCTGATCATG
36	S8I	Orai1	V102A	pEYFP YFP-Orai1	GGCTTCGCCATGGCGGCAATGGTGGAG
					CTCCACCATTGCCGCCATGGCGAAGCC
37	S8J	Orai1	H134A	pEYFP YFP-Orai1	GTGCTGGTGGCTGTGGCCCTGTTTGCGCTCAT
					ATGAGCGCAAACAGGGCCACAGCCACCAGCAC

38	S8K	Orai1	F136S	pEYFP YFP-Orai1	GGTGGCTGTGCACCTGAGTGCGCTCATGATCAGC<br>GCTGATCATGAGCGCACTCAGGTGCACAGCCACC
39	7B	Orai1	A177W V181K	pEYFP YFP-Orai1	CATCGAGCTGGCCTGGTGGTTCTCCACCAAGATCGGCACGCTGCTCT<br>AGAGCAGCGTGCCGATCTTGGTGGAGAACCACCAGGCCAGCTCGATG
40	7B	Orai1	A177F V181K	pEYFP YFP-Orai1	ATCGAGCTGGCCTGGTTCTTCTCCACCAAGATCGGCACGCTGCTC<br>GAGCAGCGTGCCGATCTTGGTGGAGAAGAACCAGGCCAGCTCGAT
41	7B	Orai1	A177L V181K	pEYFP YFP-Orai1	CACATCGAGCTGGCCTGGCTATTCTCCACCAAGATCGGCACGCTGCTCTTC<br>GAAGAGCAGCGTGCCGATCTTGGTGGAGAATAGCCAGGCCAGCTCGATGTG
42	7D	Orai1	F257W	pEYFP YFP-Orai1	GTCTTCGCCGTCCACTGGTACCGCTCACTGGTTA<br>TAACCAGTGAGCGGTACCAGTGGACGGCGAAGAC
43	7D	Orai1	F257A	pEYFP YFP-Orai1	GTCTTCGCCGTCCACGCCTACCGCTCACTGGT<br>ACCAGTGAGCGGTAGGCGTGGACGGCGAAGAC
44	7E	Orai1	F257V	pEYFP YFP-Orai1	TCTTCGCCGTCCACGTCTACCGCTCACTG<br>CAGTGAGCGGTAGACGTGGACGGCGAAGA
45	S9G	Orai1	A177F	pEYFP YFP-Orai1	CATCGAGCTGGCCTGGTTCTTCTCCACCGTCATC<br>GATGACGGTGGAGAAGAACCAGGCCAGCTCGATG
46	S9G	Orai1	A177L	pEYFP YFP-Orai1	CATCGAGCTGGCCTGGCTATTCTCCACCGTCATCG<br>CGATGACGGTGGAGAATAGCCAGGCCAGCTCGATG
47	S9G	Orai1	A177W	pEYFP YFP-Orai1	CATCGAGCTGGCCTGGTGGTTCTCCACCGTCATCG<br>CGATGACGGTGGAGAACCACCAGGCCAGCTCGATG
48	S9P	Orai1	T180F V181K	pEYFP YFP-Orai1	CTGGCCTGGGCCTTCTCCTTCAAGATCGGCACGCTGCTCTTC<br>GAAGAGCAGCGTGCCGATCTTGAAGGAGAAGGCCCAGGCCAG
49	S9P	Orai1	T180W V181K	pEYFP YFP-Orai1	GCTGGCCTGGGCCTTCTCCTGGAAGATCGGCACGCTGCTCTTCC<br>GGAAGAGCAGCGTGCCGATCTTCCAGGAGAAGGCCCAGGCCAGC
50	S9R	Orai1	T180F	pEYFP YFP-Orai1	CCTGGGCCTTCTCCTTCGTCATCGGCACGC<br>GCGTGCCGATGACGAAGGAGAAGGCCCAGG
51	S9R	Orai1	T180W	pEYFP YFP-Orai1	GCCTGGGCCTTCTCCTGGGTCATCGGCACGCTG<br>CAGCGTGCCGATGACCCAGGAGAAGGCCCAGGC
