# Supplementary material for: Water in peripheral TM-interfaces of Orai1-channels triggers pore opening
Source: Commun Biol. 2024 Nov 16;7:1522. doi: 10.1038/s42003-024-07174-6 (PMC11569263; doi:10.1038/s42003-024-07174-6)
Supplement: Supplementary file 2 — Description of Additional Supplementary Files [file 42003_2024_7174_MOESM2_ESM.pdf]

Description of Additional Supplementary Files

File name: Supplementary Movie 1

Description: The video is showing a side view of Orai1 channel during a 400ns simulation.

File name: Supplementary Movie 2

Description: The video is showing a top view of Orai1 channel during a 400ns simulation.

File name: Supplementary Data 1

Description: The file includes the statistics results.
