# Supplementary material for: Water in peripheral TM-interfaces of Orai1-channels triggers pore opening
Source: Commun Biol. 2024 Nov 16;7:1522. doi: 10.1038/s42003-024-07174-6 (PMC11569263; doi:10.1038/s42003-024-07174-6)
Supplement: Supplementary file 3 — Supplementary Data 1 [file 42003_2024_7174_MOESM3_ESM.pdf]

Supplementary Data 1: Statistics results: These results include for all statistics tests performed the type of test, the p-value, and where applicable the F values and p values of Post-hoc test.

Figure	Test	p value	F statistics	post-hoc (in case of ANOVA) p<0,05	probability	significant	
1E	Welch-ANOVA	1,24E-12	F(13, 25,98532)=31,7877	S1+O1 Orai1	0,00532	1	Patch Clamp
				V181K Orai1	5,51E-05	1	
				V181K S1+O1	0,00791	1	
				V181K + S1 Orai1	0,00566	1	
				V181K + S1 S1+O1	0,01898	1	
				V181K + S1 V181K	0,93053	0	
				V181E Orai1	0,01759	1	
				V181E S1+O1	0,85229	0	
				V181E V181K	0,00121	1	
				V181E V181K + S1	0,01001	1	
				V181E + S1 Orai1	0,02766	1	
				V181E + S1 S1+O1	0,92852	0	
				V181E + S1 V181K	0,00141	1	
				V181E + S1 V181K + S1	0,00812	1	
				V181E + S1 V181E	1	0	
				V181R Orai1	0,00698	1	
				V181R S1+O1	0,16054	0	
				V181R V181K	4,36E-04	1	
				V181R V181K + S1	0,00979	1	
				V181R V181E	0,93939	0	
				V181R V181E + S1	0,97386	0	
				V181R + S1 Orai1	0,08152	0	
				V181R + S1 S1+O1	0,25656	0	
				V181R + S1 V181K	3,31E-04	1	
				V181R + S1 V181K + S1	0,00598	1	
				V181R + S1 V181E	0,96894	0	
				V181R + S1 V181E + S1	0,98372	0	
				V181R + S1 V181R	1	0	
				V181Q Orai1	0,27467	0	

V181Q S1+O1	0,00633	1
V181Q V181K	6,80E-05	1
V181Q V181K + S1	0,00599	1
V181Q V181E	0,02387	1
V181Q V181E + S1	0,04038	1
V181Q V181R	0,0093	1
V181Q V181R + S1	0,12632	0
V181Q + S1 Orai1	0,58001	0
V181Q + S1 S1+O1	0,67114	0
V181Q + S1 V181K	0,00381	1
V181Q + S1 V181K + S1	0,00437	1
V181Q + S1 V181E	0,99508	0
V181Q + S1 V181E + S1	0,99666	0
V181Q + S1 V181R	1	0
V181Q + S1 V181R + S1	1	0
V181Q + S1 V181Q	0,6981	0
V181D Orai1	0,91834	0
V181D S1+O1	0,00413	1
V181D V181K	5,43E-05	1
V181D V181K + S1	0,0053	1
V181D V181E	0,01932	1
V181D V181E + S1	0,03823	1
V181D V181R	0,00602	1
V181D V181R + S1	0,1296	0
V181D V181Q	1	0
V181D V181Q + S1	0,71584	0
V181D + S1 Orai1	0,08913	0
V181D + S1 S1+O1	0,02816	1
V181D + S1 V181K	1,47E-04	1
V181D + S1 V181K + S1	0,0066	1
V181D + S1 V181E	0,2776	0
V181D + S1 V181E + S1	0,41961	0
V181D + S1 V181R	0,67886	0

				V181D + S1 V181R + S1	0,96331	0	
				V181D + S1 V181Q	0,15826	0	
				V181D + S1 V181Q + S1	0,99978	0	
				V181D + S1 V181D	0,17978	0	
				V181H Orai1	0,28638	0	
				V181H S1+O1	0,00636	1	
				V181H V181K	7,05E-05	1	
				V181H V181K + S1	0,00601	1	
				V181H V181E	0,02625	1	
				V181H V181E + S1	0,04668	1	
				V181H V181R	0,00985	1	
				V181H V181R + S1	0,15165	0	
				V181H V181Q	0,99982	0	
				V181H V181Q + S1	0,74668	0	
				V181H V181D	1	0	
				V181H V181D + S1	0,20837	0	
				V181H +S1 Orai1	0,02289	1	
				V181H +S1 S1+O1	0,99993	0	
				V181H +S1 V181K	0,09518	0	
				V181H +S1 V181K + S1	0,02801	1	
				V181H +S1 V181E	0,81938	0	
				V181H +S1 V181E + S1	0,86858	0	
				V181H +S1 V181R	0,36578	0	
				V181H +S1 V181R + S1	0,39224	0	
				V181H +S1 V181Q	0,03005	1	
				V181H +S1 V181Q + S1	0,60734	0	
				V181H +S1 V181D	0,02935	1	
				V181H +S1 V181D + S1	0,13073	0	
				V181H +S1 V181H	0,03331	1	
Figure	Test	p value	F statistics	post-hoc (in case of ANOVA) p<0,05	probability	significant	
1F	Welch-ANOVA	1,22E-15	F(11, 29,47925)=48,24345	O1+ S1 Orai1	0,000757757	1	Patch Clamp
				**V181G Orai1**	0,000451761	1	
				V181G O1+ S1	0,00281	1	

V181G + S1 Orai1	6,35746E-05	1
V181G + S1 O1+ S1	1	0
V181G + S1 V181G	0,000151466	1
**V181A Orai1**	0,00491	1
V181A O1+ S1	0,00266	1
V181A V181G	0,99992	0
V181A V181G + S1	8,59814E-05	1
V181A + S1 Orai1	0,000870724	1
V181A + S1 O1+ S1	0,92594	0
V181A + S1 V181G	0,00323	1
V181A + S1 V181G + S1	0,55485	0
V181A + S1 V181A	0,00291	1
V181C Orai1	0,99929	0
V181C O1+ S1	0,00082298	1
V181C V181G	0,000956508	1
V181C V181G + S1	6,57549E-05	1
V181C V181A	0,00804	1
V181C V181A + S1	0,000929837	1
V181C + S1 Orai1	0,10414	0
V181C + S1 O1+ S1	1	0
V181C + S1 V181G	0,28406	0
V181C + S1 V181G + S1	1	0
V181C + S1 V181A	0,32611	0
V181C + S1 V181A + S1	0,99998	0
V181C + S1 V181C	0,11272	0
V181S Orai1	0,02971	1
V181S O1+ S1	0,00157	1
V181S V181G	0,89485	0
V181S V181G + S1	7,82113E-05	1
V181S V181A	0,7653	0
V181S V181A + S1	0,00157	1
V181S V181C	0,05562	0
V181S V181C + S1	0,21635	0

V181S + S1 Orai1	0,00229	1
V181S + S1 O1+ S1	1	0
V181S + S1 V181G	0,01185	1
V181S + S1 V181G + S1	0,99979	0
V181S + S1 V181A	0,01418	1
V181S + S1 V181A + S1	0,9974	0
V181S + S1 V181C	0,00257	1
V181S + S1 V181C + S1	1	0
V181S + S1 V181S	0,0068	1
V181M Orai1	0,18747	0
V181M O1+ S1	0,00104	1
V181M V181G	1	0
V181M V181G + S1	0,000027996	1
V181M V181A	0,99978	0
V181M V181A + S1	0,00129	1
V181M V181C	0,26911	0
V181M V181C + S1	0,26904	0
V181M V181S	0,99995	0
V181M V181S + S1	0,00782	1
V181M + S1 Orai1	7,78316E-05	1
V181M + S1 O1+ S1	0,96427	0
V181M + S1 V181G	0,000480741	1
V181M + S1 V181G + S1	0,65454	0
V181M + S1 V181A	0,000497551	1
V181M + S1 V181A + S1	1	0
V181M + S1 V181C	8,53265E-05	1
V181M + S1 V181C + S1	1	0
V181M + S1 V181S	0,000194731	1
V181M + S1 V181S + S1	0,99941	0
V181M + S1 V181M	0,000318488	1

Figure	Test	p value	F statistics	post-hoc (in case of ANOVA) p<0,05	probability	significant	
1G	Welch-ANOVA	3,39E-10	F(11, 29,6009)=17,66366	O1+ S1 Orai1	7,42E-04	1	Patch clamp
				V181F Orai1	0,9896	0	

V181F O1+ S1	7,75E-04	1
V181F + S1 Orai1	0,99979	0
V181F + S1 O1+ S1	4,70E-04	1
V181F + S1 V181F	0,99999	0
V181W Orai1	0,15101	0
V181W O1+ S1	6,39E-04	1
V181W V181F	0,1777	0
V181W V181F + S1	0,40386	0
V181W + S1 Orai1	0,06394	0
V181W + S1 O1+ S1	0,97752	0
V181W + S1 V181F	0,06502	0
V181W + S1 V181F + S1	0,06689	0
V181W + S1 V181W	0,09731	0
**V181Y Orai1**	0,0368	1
V181Y O1+ S1	9,38E-04	1
V181Y V181F	0,04851	1
V181Y V181F + S1	0,34029	0
V181Y V181W	0,99999	0
V181Y V181W + S1	0,09057	0
V181Y + S1 Orai1	0,13279	0
V181Y + S1 O1+ S1	1	0
V181Y + S1 V181F	0,13567	0
V181Y + S1 V181F + S1	0,14209	0
V181Y + S1 V181W	0,22903	0
V181Y + S1 V181W + S1	0,99791	0
V181Y + S1 V181Y	0,20739	0
V181I Orai1	0,30347	0
V181I O1+ S1	8,15E-04	1
V181I V181F	0,59221	0
V181I V181F + S1	1	0
V181I V181W	0,26535	0
V181I V181W + S1	0,06768	0
V181I V181Y	0,09439	0

				V181I V181Y + S1	0,14282	0	
				V181I +S1 Orai1	0,06003	0	
				V181I +S1 O1+ S1	0,9272	0	
				V181I +S1 V181F	0,06248	0	
				V181I +S1 V181F + S1	0,0682	0	
				V181I +S1 V181W	0,1694	0	
				V181I +S1 V181W + S1	0,74989	0	
				V181I +S1 V181Y	0,1389	0	
				V181I +S1 V181Y + S1	0,99778	0	
				V181I +S1 V181I	0,06877	0	
				V181L Orai1	0,97255	0	
				V181L O1+ S1	7,79E-04	1	
				V181L V181F	1	0	
				V181L V181F + S1	1	0	
				V181L V181W	0,17802	0	
				V181L V181W + S1	0,06505	0	
				V181L V181Y	0,04892	1	
				V181L V181Y + S1	0,13573	0	
				V181L V181I	0,53579	0	
				V181L V181I +S1	0,06254	0	
				V181L + S1 Orai1	0,02854	1	
				V181L + S1 O1+ S1	0,99999	0	
				V181L + S1 V181F	0,02914	1	
				V181L + S1 V181F + S1	0,02904	1	
				V181L + S1 V181W	0,04526	1	
				V181L + S1 V181W + S1	0,99936	0	
				V181L + S1 V181Y	0,04278	1	
				V181L + S1 V181Y + S1	1	0	
				V181L + S1 V181I	0,03056	1	
				V181L + S1 V181I +S1	0,90873	0	
				V181L + S1 V181L	0,02916	1	
Figure	Test	p value	F statistics	post-hoc (in case of ANOVA) p<0,05	probability	significant	
1H	OneWay ANOVA	0,02186	F(2,15)=4.98653	**V181K WT**	0,00743	1	Patch clamp

				V181K +S1 WT	0,33672	0	
				V181K +S1 V181K	0,05304	0	
Figure	Test	p value	statistics	post-hoc (in case of ANOVA) p<0,05	probability	significant	
1I	OneWay ANOVA	1,25E-01	F(2,17)=2.35407	Orai1 V181A WT	2,41E-01	0	Patch clamp
				Orai1 V181A + S1 WT	0,39708	0	
				**Orai1 V181A + S1 Orai1 V181A**	4,67E-02	1	
Figure	Test	p value	statistics	post-hoc (in case of ANOVA) p<0,05	probability	significant	
1J	OneWay ANOVA	2,88E-05	F(2,21)=17.92006	**Orai1 V181W Orai1**	0,00354	1	Patch clamp
				Orai1 V181W + STIM1 Orai1	0,11703	0	
				**Orai1 V181W + STIM1 Orai1 V181W**	7,44E-06	1	
Figure	Test	p value	statistics	post-hoc (in case of ANOVA) p<0,05	probability	significant	
1K	Kruskal-Wallis ANC	5,94E-37	Chi-Quadrat: 171.53307 DF:	**WT V181K**	<0.0001	1	NFAT
				WT V181A	0,04333	0	
				WT V181W	0,01336	0	
				V181K V181A	<0.0001	1	
				V181K V181W	<0.0001	1	
				V181A V181W	1	0	
Figure	Test	p value	statistics	post-hoc (in case of ANOVA) p<0,05	probability	significant	
2C	Welch-ANOVA	4,50E-12	F(7, 15.90645)=102.00394	O1 + S1 Orai1	1,93E-06	1	Patch clamp
				**A254K Orai1**	0,00242	1	
				A254K O1 + S1	0,9958	0	
				A254K +S1 Orai1	0,04955	1	
				A254K +S1 O1 + S1	0,20596	0	
				A254K +S1 A254K	0,17767	0	
				**A254E Orai1**	0,04015	1	
				A254E O1 + S1	0,99999	0	
				A254E A254K	0,99848	0	
				A254E A254K +S1	0,23392	0	
				A254E + S1 Orai1	0,00392	1	
				A254E + S1 O1 + S1	0,93388	0	
				A254E + S1 A254K	0,87036	0	
				A254E + S1 A254K +S1	0,28591	0	
				A254E + S1 A254E	0,99939	0	

				**A254Q Orai1**	0,0041	1	
				A254Q O1 + S1	0,62167	0	
				A254Q A254K	0,56998	0	
				A254Q A254K +S1	0,3437	0	
				A254Q A254E	0,97576	0	
				A254Q A254E + S1	0,99949	0	
				A254Q +S1 Orai1	0,12833	0	
				A254Q +S1 O1 + S1	0,6747	0	
				A254Q +S1 A254K	0,61605	0	
				A254Q +S1 A254K +S1	0,96871	0	
				A254Q +S1 A254E	0,77337	0	
				A254Q +S1 A254E + S1	0,85538	0	
				A254Q +S1 A254Q	0,92098	0	
Figure	Test	p value	statistics	post-hoc (in case of ANOVA) p<0,05	probability	significant	
2D	OneWay ANOVA	0,0086	F(2,16)=6.49779	**A254K WT**	0,02933	1	Patch clamp
				A254K_S1 WT	0,28754	0	
				**A254K_S1 A254K**	0,0034	1	
Figure	Test	p value	statistics	post-hoc (in case of ANOVA) p<0,05	probability	significant	
2E	Kruskal-Wallis ANC	1,78E-38	Chi-Quadrat: 178.58643 DF:	**WT A254K**	<0.0001	1	NFAT
				**WT A254E**	<0.0001	1	
				**WT A254Q**	<0.0001	1	
				A254K A254E	1	0	
				A254K A254Q	1	0	
				A254E A254Q	1	0	
Figure	Test	p value	statistics	post-hoc (in case of ANOVA) p<0,05	probability	significant	
6C	OneWay ANOVA	9,24E-06	F(2,18)=23.63042	**V181K F253A V181K**	0,00269	1	Patch clamp
				**V181K F253W V181K**	0,00147	1	
				V181K F253W V181K F253A	1,98E-06	1	
Figure	Test	p value	statistics	post-hoc (in case of ANOVA) p<0,05	probability	significant	
6E	Welch-ANOVA	1,56E-07	F(4,13.93785)=40.26511	**V181K C143A V181K**	9,08E-07	1	Patch clamp
				V181K C143F V181K	0,99981	0	
				V181K C143F V181K C143A	0,03868	1	
				V181K C143L V181K	0,99936	0	

				V181K C143L V181K C143A	0,02624	1	
				V181K C143L V181K C143F	1	0	
				V181K C143W V181K	0,6531	0	
				V181K C143W V181K C143A	0,05674	0	
				V181K C143W V181K C143F	0,81017	0	
				V181K C143W V181K C143L	0,81178	0	
Figure	Test	p value	statistics	post-hoc (in case of ANOVA) p<0,05	probability	significant	
7C	Welch-ANOVA	1,74E-07	F(3,12.39836)=59.76304	**V181K A177F V181K**	0,00166	1	Patch clamp
				V181K A177L V181K	0,15475	0	
				V181K A177L V181K A177F	0,88905	0	
				**V181K A177W V181K**	4,45E-06	1	
				V181K A177W V181K A177F	0,00527	1	
				V181K A177W V181K A177L	0,02671	1	
Figure	Test	p value	statistics	post-hoc (in case of ANOVA) p<0,05	probability	significant	
7E	Welch-ANOVA	5,75E-08	F(7,17.25052)=26.59988	V181K F257A V181K	0,99902	0	Patch clamp
				**V181K F257W V181K**	0,00388	1	
				V181K F257W V181K F257A	0,04742	1	
				**V181K A177W F257W V181K**	0,00398	1	
				V181K A177W F257W V181K F257A	0,04788	1	
				V181K A177W F257W V181K F257W	1	0	
				V181R V181K	0,02282	1	
				V181R V181K F257A	0,3318	0	
				V181R V181K F257W	0,00625	1	
				V181R V181K A177W F257W	0,00688	1	
				V181R F257A V181K	0,99999	0	
				V181R F257A V181K F257A	0,99999	0	
				V181R F257A V181K F257W	0,03145	1	
				V181R F257A V181K A177W F257W	0,03178	1	
				V181R F257A V181R	0,2187	0	
				V181R F257V V181K	0,99957	0	
				V181R F257V V181K F257A	0,98906	0	
				V181R F257V V181K F257W	0,03928	1	
				V181R F257V V181K A177W F257W	0,03959	1	

V181R F257V V181R	0,16276	0
V181R F257V V181R F257A	0,99843	0
V181R F257W V181K	0,00352	1
V181R F257W V181K F257A	0,04094	1
V181R F257W V181K F257W	0,24267	0
V181R F257W V181K A177W F257W	0,03194	1
**V181R F257W V181R**	0,0052	1
V181R F257W V181R F257A	0,0273	1
V181R F257W V181R F257V	0,03541	1

Figure	Test	p value	F statistics	post-hoc (in case of ANOVA) p<0,05	probability	significant	
S2A	Kruskal-Wallis ANOVA	4,14E-55	Chi-Quadrat: 264.54915 DF: 5	**WT V181K**	<0.0001	1	NFAT
				**WT V181E**	<0.0001	1	
				**WT V181R**	<0.0001	1	
				WT V181Q	1	0	
				WT V181H	0,0277	0	
				V181K V181E	1	0	
				V181K V181R	1	0	
				V181K V181Q	<0.0001	1	
				V181K V181H	<0.0001	1	
				V181E V181R	1	0	
				V181E V181Q	<0.0001	1	
				V181E V181H	1,22E-04	1	
				V181R V181Q	<0.0001	1	
				V181R V181H	<0.0001	1	
				V181Q V181H	0,00665	1	
Figure	Test	p value	statistics	post-hoc (in case of ANOVA) p<0,05	probability	significant	
S2B	Kruskal-Wallis ANOVA	5,97E-17	Chi-Quadrat: 78.65163 DF:3	**WT V181G**	<0.0001	1	NFAT
				**WT V181A**	0,00156	1	
				**WT V181S**	4,37E-04	1	
				V181G V181A	<0.0001	1	
				V181G V181S	1,25E-04	1	
				V181A V181S	1	0	
Figure	Test	p value	statistics	post-hoc (in case of ANOVA) p<0,05	probability	significant	
S2C	Kruskal-Wallis ANOVA	7,91E-21	Chi-Quadrat: 103.91225 DF: 5	WT V181F	0,42632	0	NFAT
				**WT V181W**	5,53E-04	1	
				WT V181I	1	0	
				WT V181L	0,72791	0	
				**WT V181Y**	<0.0001	1	
				V181F V181W	<0.0001	1	
				V181F V181I	1	0	
				V181F V181L	1	0	
				V181F V181Y	<0.0001	1	
				V181W V181I	0,00149	1	
				V181W V181L	<0.0001	1	

				V181W V181Y	<0.0001	1	
				V181I V181L	1	0	
				V181I V181Y	<0.0001	1	
				V181L V181Y	<0.0001	1	
Figure	Test	p value	statistics	post-hoc (in case of ANOVA) p<0,05	probability	significant	
S2E	Kruskal-Wallis ANOVA	1,27E-28	Chi-Quadrat: 147.64867 DF: 7	WT start WT end	<0.0001	1	NFAT
				WT start V181K start	<0.0001	1	
				WT start V181K end	<0.0001	1	
				WT start V181E start	<0.0001	1	
				WT start V181E end	<0.0001	1	
				WT start V181R start	<0.0001	1	
				WT start V181R end	<0.0001	1	
				WT end V181K start	0,00638	1	
				WT end V181K end	1	0	
				WT end V181E start	0,06659	0	
				WT end V181E end	1	0	
				WT end V181R start	0,01529	0	
				WT end V181R end	1	0	
				V181K start V181K end	0,1997	0	
				V181K start V181E start	1	0	
				V181K start V181E end	0,31435	0	
				V181K start V181R start	1	0	
				V181K start V181R end	0,1997	0	
				V181K end V181E start	0,55403	0	
				V181K end V181E end	1	0	
				V181K end V181R start	0,2244	0	
				V181K end V181R end	1	0	
				V181E start V181E end	0,75743	0	
				V181E start V181R start	1	0	
				V181E start V181R end	0,55403	0	
				V181E end V181R start	0,33292	0	
				V181E end V181R end	1	0	
				V181R start V181R end	0,2244	0	
Figure	Test	p value	statistics	post-hoc (in case of ANOVA) p<0,05	probability	significant	
S2F	Kruskal-Wallis ANOVA	2,45E-20	Chi-Quadrat: 101.5837 DF:5	WT start WT end	<0.0001	1	NFAT
				WT start V181A start	0,62476	0	
				WT start V181A end	<0.0001	1	

				WT start V181G start	0,02616	0	
				WT start V181G end	<0.0001	1	
				WT end V181A start	<0.0001	1	
				WT end V181A end	1	0	
				WT end V181G start	<0.0001	1	
				WT end V181G end	1	0	
				V181A start V181A end	<0.0001	1	
				V181A start V181G start	1	0	
				V181A start V181G end	<0.0001	1	
				V181A end V181G start	0,00202	1	
				V181A end V181G end	1	0	
				V181G start V181G end	0,00202	1	

Figure	Test	p value	statistics	post-hoc (in case of ANOVA) p<0,05	probability	significant	
S2G	Kruskal-Wallis ANOVA	9,19E-26	Chi-Quadrat: 133.99446 DF:7	WT start WT end	<0.0001	1	NFAT
				WT start V181L start	1	0	
				WT start V181L end	<0.0001	1	
				WT start V181W start	1	0	
				WT start V181W end	<0.0001	1	
				WT start V181Y start	1	0	
				WT start V181Y end	<0.0001	1	
				WT end V181L start	<0.0001	1	
				WT end V181L end	1	0	
				WT end V181W start	<0.0001	1	
				WT end V181W end	1	0	
				WT end V181Y start	<0.0001	1	
				WT end V181Y end	1	0	
				V181L start V181L end	<0.0001	1	
				V181L start V181W start	1	0	
				V181L start V181W end	<0.0001	1	
				V181L start V181Y start	1	0	
				V181L start V181Y end	<0.0001	1	
				V181L end V181W start	<0.0001	1	
				V181L end V181W end	1	0	
				V181L end V181Y start	<0.0001	1	
				V181L end V181Y end	1	0	
				V181W start V181W end	<0.0001	1	
				V181W start V181Y start	1	0	

				V181W start V181Y end	<0.0001	1	
				V181W end V181Y start	<0.0001	1	
				V181W end V181Y end	1	0	
				V181Y start V181Y end	<0.0001	1	
Figure	Test	p value	statistics	post-hoc (in case of ANOVA) p<0,05	probability	significant	
S2H	Kruskal-Wallis ANOVA	0,00647	Chi-Quadrat: 95.55792 DF:7	WT start +STIM WT end + STIM	0,00647	1	NFAT
				WT start +STIM V181K start + STIM	3,19E-04	1	
				WT start +STIM V181K end + STIM	<0.0001	1	
				WT start +STIM V181E start + STIM	<0.0001	1	
				WT start +STIM V181E end + STIM	<0.0001	1	
				WT start +STIM V181R start + STIM	5,50E-04	1	
				WT start +STIM V181R end + STIM	<0.0001	1	
				WT end + STIM V181K start + STIM	1	0	
				WT end + STIM V181K end + STIM	0,16698	0	
				WT end + STIM V181E start + STIM	1	0	
				WT end + STIM V181E end + STIM	0,13332	0	
				WT end + STIM V181R start + STIM	1	0	
				WT end + STIM V181R end + STIM	0,71062	0	
				V181K start + STIM V181K end + STIM	0,29403	0	
				V181K start + STIM V181E start + STIM	1	0	
				V181K start + STIM V181E end + STIM	0,23439	0	
				V181K start + STIM V181R start + STIM	1	0	
				V181K start + STIM V181R end + STIM	1	0	
				V181K end + STIM V181E start + STIM	1	0	
				V181K end + STIM V181E end + STIM	1	0	
				V181K end + STIM V181R start + STIM	1	0	
				V181K end + STIM V181R end + STIM	1	0	
				V181E start + STIM V181E end + STIM	1	0	
				V181E start + STIM V181R start + STIM	1	0	
				V181E start + STIM V181R end + STIM	1	0	
				V181E end + STIM V181R start + STIM	0,89341	0	
				V181E end + STIM V181R end + STIM	1	0	
				V181R start + STIM V181R end + STIM	1	0	
Figure	Test	p value	statistics	post-hoc (in case of ANOVA) p<0,05	probability	significant	
S2I	Kruskal-Wallis ANOVA	3,21E-20	Chi-Quadrat: 101.0299 DF:5	WT start +STIM WT end + STIM	<0.0001	1	NFAT
				WT start +STIM V181A start + STIM	1	0	
				WT start +STIM V181A end + STIM	<0.0001	1	

Figure	Test	p value	statistics	post-hoc (in case of ANOVA) p<0,05	probability	significant	
				WT start +STIM V181G start + STIM	0,91143	0	
				WT start +STIM V181G end + STIM	<0.0001	1	
				WT end + STIM V181A start + STIM	0,00538	1	
				WT end + STIM V181A end + STIM	1	0	
				WT end + STIM V181G start + STIM	0,00915	1	
				WT end + STIM V181G end + STIM	1	0	
				V181A start + STIM V181A end + STIM	<0.0001	1	
				V181A start + STIM V181G start + STIM	1	0	
				V181A start + STIM V181G end + STIM	<0.0001	1	
				V181A end + STIM V181G start + STIM	<0.0001	1	
				V181A end + STIM V181G end + STIM	1	0	
				V181G start + STIM V181G end + STIM	<0.0001	1	
Figure	Test	p value	statistics	post-hoc (in case of ANOVA) p<0,05	probability	significant	
S2J	Kruskal-Wallis ANOVA	4,17E-26	Chi-Quadrat: 135.6363 DF:7	WT start +STIM WT end + STIM	<0.0001	1	NFAT
				WT start +STIM V181L start + STIM	1	0	
				WT start +STIM V181L end + STIM	<0.0001	1	
				WT start +STIM V181W start + STIM	1	0	
				WT start +STIM V181W end + STIM	<0.0001	1	
				WT start +STIM V181Y start + STIM	1	0	
				WT start +STIM V181Y end + STIM	<0.0001	1	
				WT end + STIM V181L start + STIM	<0.0001	1	
				WT end + STIM V181L end + STIM	1	0	
				WT end + STIM V181W start + STIM	0,0272	0	
				WT end + STIM V181W end + STIM	1	0	
				WT end + STIM V181Y start + STIM	0,00353	1	
				WT end + STIM V181Y end + STIM	1	0	
				V181L start + STIM V181L end + STIM	<0.0001	1	
				V181L start + STIM V181W start + STIM	0,18465	0	
				V181L start + STIM V181W end + STIM	<0.0001	1	
				V181L start + STIM V181Y start + STIM	0,97387	0	
				V181L start + STIM V181Y end + STIM	<0.0001	1	
				V181L end + STIM V181W start + STIM	0,00724	1	
				V181L end + STIM V181W end + STIM	1	0	
				V181L end + STIM V181Y start + STIM	5,90E-04	1	
				V181L end + STIM V181Y end + STIM	1	0	
				V181W start + STIM V181W end + STIM	<0.0001	1	
				V181W start + STIM V181Y start + STIM	1	0	

				V181W start + STIM V181Y end + STIM	1,67E-04	1	
				V181W end + STIM V181Y start + STIM	<0.0001	1	
				V181W end + STIM V181Y end + STIM	1	0	
				V181Y start + STIM V181Y end + STIM	<0.0001	1	
Figure	Test	p value	statistics	post-hoc (in case of ANOVA) p<0,05	probability	significant	
S3A	Welch ANOVA	1.36754E-7	F(4, 68.94889)=12.27019	V181D WT	1.11192E-6	1	FRET
				V181E WT	0.76235	0	
				V181E V181D	0.0162	1	
				V181K WT	0.93137	0	
				V181K V181D	1.87043E-6	1	
				V181K V181E	0.47211	0	
				V181R WT	0.74467	0	
				V181R V181D	4.17835E-4	1	
				V181R V181E	0.99939	0	
				V181R V181K	0.41051	0	
Figure	Test	p value	statistics	post-hoc (in case of ANOVA) p<0,05	probability	significant	
S3B	Welch ANOVA	0.13587	F(2, 42.75077)=2.09167	V181A WT	0.46821	0	FRET
				V181G WT	0.80484	0	
				V181G V181A	0.13096	0	
Figure	Test	p value	statistics	post-hoc (in case of ANOVA) p<0,05	probability	significant	
S3C	Welch ANOVA	0.3056	F(4, 69.13217)=1.2315	V181F WT	0.78965	0	FRET
				V181L WT	0.65639	0	
				V181L V181F	0.99991	0	
				V181W WT	0.99989	0	
				V181W V181F	0.92499	0	
				V181W V181L	0.86638	0	
				V181Y WT	0.9995	0	
				V181Y V181F	0.54168	0	
				V181Y V181L	0.33687	0	
				V181Y V181W	0.99691	0	
Figure	Test	p value	statistics	post-hoc (in case of ANOVA) p<0,05	probability	significant	
S3E	Welch ANOVA	1.2116E-4	F(2, 34.0531)=11.89613	A254E WT	1.44178E-4	1	
				A254K WT	3.31781E-4	1	
				A254K A254E	0.94748	0	
Figure	Test	p value	statistics	post-hoc (in case of ANOVA) p<0,05	probability	significant	
S4A	Welch-ANOVA	0	F(4, 125.89945)=158.07507	Orai1-CFP-YFP V181A (TransFectin 1ug) Orai1-CFP-YFP (Trar	0,48711	0	FRET
				**Orai1-CFP-YFP V181E (TransFectin 1ug) Orai1-CFP-YFP (Tra**	0	1	

				Orai1-CFP-YFP V181E (TransFectin 1ug) Orai1-CFP-YFP V181	0	1	
				**Orai1-CFP-YFP V181K (TransFectin 1ug) Orai1-CFP-YFP (Tra**	2,41E-08	1	
				Orai1-CFP-YFP V181K (TransFectin 1ug) Orai1-CFP-YFP V181	0	1	
				Orai1-CFP-YFP V181K (TransFectin 1ug) Orai1-CFP-YFP V181	0	1	
				**Orai1-CFP-YFP V181R (TransFectin 1ug) Orai1-CFP-YFP (Tra**	0	1	
				Orai1-CFP-YFP V181R (TransFectin 1ug) Orai1-CFP-YFP V181	0	1	
				Orai1-CFP-YFP V181R (TransFectin 1ug) Orai1-CFP-YFP V181	0,01226	1	
				Orai1-CFP-YFP V181R (TransFectin 1ug) Orai1-CFP-YFP V181	1,13E-06	1	
Figure	Test	p value	statistics	post-hoc (in case of ANOVA) p<0,05	probability	significant	
S4E	Welch-ANOVA	1,77E-14	F(13,47.60112)=18.6681	**O1+ S1 O1**	0,00319	1	Patch clamp
				A177K O1	0,44757	0	
				A177K O1+ S1	0,00388	1	
				A177K+S1 O1	0,0062	1	
				A177K+S1 O1+ S1	1	0	
				**A177K+S1 A177K**	0,00758	1	
				L185K_F250K O1	0,98134	0	
				L185K_F250K O1+ S1	0,00339	1	
				L185K_F250K A177K	0,9052	0	
				L185K_F250K A177K+S1	0,0066	1	
				L185K_F250K+S1 O1	0,22543	0	
				L185K_F250K+S1 O1+ S1	0,00424	1	
				L185K_F250K+S1 A177K	0,9999	0	
				L185K_F250K+S1 A177K+S1	0,00827	1	
				L185K_F250K+S1 L185K_F250K	0,56324	0	
				F187K O1	0,04334	1	
				F187K O1+ S1	0,00902	1	
				F187K A177K	0,13852	0	
				F187K A177K+S1	0,0178	1	
				F187K L185K_F250K	0,06024	0	
				F187K L185K_F250K+S1	0,23762	0	
				F187K+S1 O1	0,11047	0	
				F187K+S1 O1+ S1	0,00724	1	
				F187K+S1 A177K	0,35983	0	
				F187K+S1 A177K+S1	0,0145	1	
				F187K+S1 L185K_F250K	0,15727	0	
				F187K+S1 L185K_F250K+S1	0,56187	0	
				F187K+S1 F187K	0,99999	0	

A189K O1	0,99961	0
A189K O1+ S1	0,00336	1
A189K A177K	0,98965	0
A189K A177K+S1	0,0066	1
A189K L185K_F250K	1	0
A189K L185K_F250K+S1	0,82893	0
A189K F187K	0,06479	0
A189K F187K+S1	0,17911	0
A189K+S1 O1	0,00457	1
A189K+S1 O1+ S1	0,74481	0
A189K+S1 A177K	0,00514	1
A189K+S1 A177K+S1	0,87892	0
A189K+S1 L185K_F250K	0,00474	1
A189K+S1 L185K_F250K+S1	0,00541	1
A189K+S1 F187K	0,00829	1
A189K+S1 F187K+S1	0,00735	1
**A189K+S1 A189K**	0,00473	1
L193K O1	0,99873	0
L193K O1+ S1	0,0034	1
L193K A177K	0,99502	0
L193K A177K+S1	0,00667	1
L193K L185K_F250K	1	0
L193K L185K_F250K+S1	0,86626	0
L193K F187K	0,06847	0
L193K F187K+S1	0,18909	0
L193K A189K	1	0
L193K A189K+S1	0,00476	1
L193K+S1 O1	8,96E-04	1
L193K+S1 O1+ S1	0,1466	0
L193K+S1 A177K	0,00139	1
L193K+S1 A177K+S1	0,20981	0
L193K+S1 L185K_F250K	0,00103	1
L193K+S1 L185K_F250K+S1	0,00172	1
L193K+S1 F187K	0,02096	1
L193K+S1 F187K+S1	0,01035	1
L193K+S1 A189K	9,34E-04	1
L193K+S1 A189K+S1	0,0359	1

				**L193K+S1 L193K**	9,62E-04	1	
				C195K O1	0,94221	0	
				C195K O1+ S1	0,00369	1	
				C195K A177K	1	0	
				C195K A177K+S1	0,00731	1	
				C195K L185K_F250K	0,9988	0	
				C195K L185K_F250K+S1	0,99985	0	
				C195K F187K	0,14009	0	
				C195K F187K+S1	0,37306	0	
				C195K A189K	0,99988	0	
				C195K A189K+S1	0,00501	1	
				C195K L193K	0,99996	0	
				C195K L193K+S1	0,00112	1	
				C195K+S1 O1	5,78E-04	1	
				C195K+S1 O1+ S1	0,98082	0	
				C195K+S1 A177K	7,56E-04	1	
				C195K+S1 A177K+S1	0,97259	0	
				C195K+S1 L185K_F250K	6,30E-04	1	
				C195K+S1 L185K_F250K+S1	8,55E-04	1	
				C195K+S1 F187K	0,00263	1	
				C195K+S1 F187K+S1	0,00182	1	
				C195K+S1 A189K	6,02E-04	1	
				C195K+S1 A189K+S1	0,22106	0	
				C195K+S1 L193K	6,13E-04	1	
				C195K+S1 L193K+S1	0,33826	0	
				**C195K+S1 C195K**	6,70E-04	1	

Figure	Test	p value	statistics	post-hoc (in case of ANOVA) p<0,05	probability	significant	
S4F	Welch-ANOVA	1,78E-06	F(3, 15.16165)=28.85288	**O1+S1 O1**	6,99E-05	1	Patch clamp
				F253K O1	0,33559	0	
				F253K O1+S1	8,12E-05	1	
				F253K+S1 O1	0,00367	1	
				F253K+S1 O1+S1	0,09244	0	
				**F253K+S1 F253K**	0,00452	1	

Figure	Test	p value	statistics	post-hoc (in case of ANOVA) p<0,05	probability	significant	
S8B	OneWay ANOVA	0,29343	F(2, 21)=1.30056	V181K F253A + S1 V181K + S1	0,12662	0	Patch clamp
				V181K F253W+ S1 V181K + S1	0,58154	0	
				V181K F253W+ S1 V181K F253A + S1	0,34854	0	

Figure	Test	p value	statistics	post-hoc (in case of ANOVA) p<0,05	probability	significant	
S8E	Welch-ANOVA	5,09E-10	F(4, 17.84717)=57.67739	**V181K C143A + S1 V181K + S1**	1,56E-06	1	Patch clamp
				V181K C143F + S1 V181K + S1	0,78678	0	
				V181K C143F + S1 V181K C143A + S1	0,00248	1	
				V181K C143L + S1 V181K + S1	0,88671	0	
				V181K C143L + S1 V181K C143A + S1	1,17E-05	1	
				V181K C143L + S1 V181K C143F + S1	0,99094	0	
				V181K C143W + S1 V181K + S1	0,9999	0	
				V181K C143W + S1 V181K C143A + S1	0,06983	0	
				V181K C143W + S1 V181K C143F + S1	0,94802	0	
				V181K C143W + S1 V181K C143L + S1	0,98367	0	

Figure	Test	p value	statistics	post-hoc (in case of ANOVA) p<0,05	probability	significant	
S8G	Welch-ANOVA	9,32E-09	F(5, 14.75052)=48.90118	O1+S1 O1	4,16E-05	1	Patch clamp
				O1 F253A O1	0,85831	0	
				O1 F253A O1+S1	7,31E-06	1	
				O1 F253A+S1 O1	0,00199	1	
				O1 F253A+S1 O1+S1	0,9138	0	
				O1 F253A+S1 O1 F253A	0,00409	1	
				O1 F253W O1	0,09346	0	
				O1 F253W O1+S1	9,30E-06	1	
				O1 F253W O1 F253A	0,9956	0	
				O1 F253W O1 F253A+S1	0,00564	1	
				O1 F253W+S1 O1	0,03915	1	
				O1 F253W+S1 O1+S1	0,93811	0	
				O1 F253W+S1 O1 F253A	0,0471	1	
				O1 F253W+S1 O1 F253A+S1	0,77358	0	
				O1 F253W+S1 O1 F253W	0,05613	0	

Figure	Test	p value	statistics	post-hoc (in case of ANOVA) p<0,05	probability	significant	
S8H	Welch-ANOVA	2,71E-08	F(9, 23.44958)=17.51168	O1+S1 O1	3,29E-04	1	Patch clamp
				C143A O1	0,9113	0	
				C143A O1+S1	6,15E-04	1	
				C143A + S1 O1	0,30052	0	
				C143A + S1 O1+S1	0,98007	0	
				C143A + S1 C143A	0,4124	0	
				C143F O1	0,97513	0	
				C143F O1+S1	6,89E-04	1	
				C143F C143A	0,21885	0	

				C143F C143A + S1	0,24601	0
				C143F + S1 O1	0,61698	0
				C143F + S1 O1+S1	0,99916	0
				C143F + S1 C143A	0,71754	0
				C143F + S1 C143A + S1	1	0
				C143F + S1 C143F	0,55732	0
				C143L O1	0,82944	0
				C143L O1+S1	5,95E-04	1
				C143L C143A	0,99999	0
				C143L C143A + S1	0,46072	0
				C143L C143F	0,31268	0
				C143L C143F + S1	0,75277	0
				C143L + S1 O1	0,08535	0
				C143L + S1 O1+S1	0,01501	1
				C143L + S1 C143A	0,23624	0
				C143L + S1 C143A + S1	0,93564	0
				C143L + S1 C143F	0,05024	0
				C143L + S1 C143F + S1	0,9799	0
				C143L + S1 C143L	0,36703	0
				C143W O1	0,37045	0
				C143W O1+S1	0,99999	0
				C143W C143A	0,48426	0
				C143W C143A + S1	1	0
				C143W C143F	0,30975	0
				C143W C143F + S1	1	0
				C143W C143L	0,5291	0
				C143W C143L + S1	0,9195	0
				C143W + S1 O1	0,01533	1
				C143W + S1 O1+S1	0,11824	0
				C143W + S1 C143A	0,01785	1
				C143W + S1 C143A + S1	0,06804	0
				C143W + S1 C143F	0,0144	1
				C143W + S1 C143F + S1	0,11877	0
				C143W + S1 C143L	0,01851	1
				C143W + S1 C143L + S1	0,03006	1
				C143W + S1 C143W	0,12029	0
Figure	Test	p value	statistics	post-hoc (in case of ANOVA) p<0,05	probability	significant

Figure	Test	p value	statistics	post-hoc (in case of ANOVA) p<0,05	probability	significant	
S8I	OneWay ANOVA	0,04085	F(2,18)=3,83965	V102A F253A V102A	0,61611	0	Patch clamp
				V102A F253W V102A	0,01425	1	
				V102A F253W V102A F253A	0,03664	1	
Figure	Test	p value	statistics	post-hoc (in case of ANOVA) p<0,05	probability	significant	
S8J	OneWay ANOVA	0,25901	F(2,27)=1,4208	H134A F253A H134A	0,1034	0	Patch clamp
				H134A F253W H134A	0,52354	0	
				H134A F253W H134A F253A	0,37268	0	
Figure	Test	p value	statistics	post-hoc (in case of ANOVA) p<0,05	probability	significant	
S8K	Mann-Whitney test	NS(0,45191)					Patch clamp
Figure	Test	p value	statistics	post-hoc (in case of ANOVA) p<0,05	probability	significant	
S8L	Welch-ANOVA	0,04188	F(2, 10,81095)=4,29288	V102A C143A V102A	0,03872	1	Patch clamp
				V102A C143W V102A	0,28822	0	
				V102A C143W V102A C143A	0,11641	0	
Figure	Test	p value	statistics	post-hoc (in case of ANOVA) p<0,05	probability	significant	
S8M	OneWay ANOVA	0,0316	F(2,22)=4,0584	H134A C143A H134A	0,24501	0	Patch clamp
				H134A C143W H134A	0,00975	1	
				H134A C143W H134A C143A	0,24584	0	
Figure	Test	p value	statistics	post-hoc (in case of ANOVA) p<0,05	probability	significant	
S8N	Mann-Whitney test	S(0,04539)					Patch clamp
Figure	Test	p value	statistics	post-hoc (in case of ANOVA) p<0,05	probability	significant	
S9B	Welch-ANOVA	1,79E-07	F(3,12.89888)=51.47713	V181K A177F + S1 V181K + S1	0,12011	0	Patch clamp
				V181K A177L + S1 V181K + S1	0,4453	0	
				V181K A177L + S1 V181K A177F + S1	0,98301	0	
				**V181K A177W + S1 V181K + S1**	1,56E-05	1	
				V181K A177W + S1 V181K A177F + S1	0,00374	1	
				V181K A177W + S1 V181K A177L + S1	0,00937	1	
Figure	Test	p value	statistics	post-hoc (in case of ANOVA) p<0,05	probability	significant	
S9E	Welch-ANOVA	4,88E-06	F(7,19.15134)=12.94628	V181K F257A + S1 V181K + S1	1	0	Patch clamp
				V181K F257W+ S1 V181K + S1	0,05726	0	
				V181K F257W+ S1 V181K F257A + S1	0,22775	0	
				V181K A177W F257W + S1 V181K + S1	0,00198	1	
				V181K A177W F257W + S1 V181K F257A + S1	0,01496	1	
				V181K A177W F257W + S1 V181K F257W+ S1	0,09757	0	
				V181R + S1 V181K + S1	0,00566	1	
				V181R + S1 V181K F257A + S1	0,03093	1	
				V181R + S1 V181K F257W+ S1	0,29747	0	
				V181R + S1 V181K A177W F257W + S1	0,86416	0	

				V181R F257A V181K + S1	0,60928	0	
				V181R F257A V181K F257A + S1	0,83102	0	
				V181R F257A V181K F257W+ S1	0,87276	0	
				V181R F257A V181K A177W F257W + S1	0,05192	0	
				V181R F257A V181R + S1	0,12242	0	
				V181R F257V V181K + S1	0,94834	0	
				V181R F257V V181K F257A + S1	0,98701	0	
				V181R F257V V181K F257W+ S1	0,60563	0	
				V181R F257V V181K A177W F257W + S1	0,05249	0	
				V181R F257V V181R + S1	0,1025	0	
				V181R F257V V181R F257A	0,99843	0	
				V181R F257W + S1 V181K + S1	0,00433	1	
				V181R F257W + S1 V181K F257A + S1	0,02727	1	
				V181R F257W + S1 V181K F257W+ S1	0,26572	0	
				V181R F257W + S1 V181K A177W F257W + S1	0,95722	0	
				V181R F257W + S1 V181R + S1	1	0	
				V181R F257W + S1 V181R F257A	0,10852	0	
				V181R F257W + S1 V181R F257V	0,09188	0	

Figure	Test	p value	statistics	post-hoc (in case of ANOVA) p<0,05	probability	significant	
S9G	Welch-ANOVA	8,70E-10	F(7, 23.76785)=26.32741	O1+S1 O1	5,72E-04	1	Patch clamp
				A177F O1	0,99962	0	
				A177F O1+S1	5,49E-04	1	
				A177F +S1 O1	0,31248	0	
				A177F +S1 O1+S1	2,82E-04	1	
				A177F +S1 A177F	0,27429	0	
				A177L O1	0,51216	0	
				A177L O1+S1	5,00E-04	1	
				A177L A177F	0,45092	0	
				A177L A177F +S1	0,94344	0	
				A177L +S1 O1	0,23802	0	
				A177L +S1 O1+S1	0,94633	0	
				A177L +S1 A177F	0,23544	0	
				A177L +S1 A177F +S1	0,30309	0	
				A177L +S1 A177L	0,26643	0	
				**A177W O1**	0,00542	1	
				A177W O1+S1	5,09E-04	1	

				A177W A177F	0,00413	1	
				A177W A177F +S1	0,99805	0	
				A177W A177L	0,35767	0	
				A177W A177L +S1	0,32738	0	
				A177W +S1 O1	0,0025	1	
				A177W +S1 O1+S1	0,00135	1	
				A177W +S1 A177F	0,00229	1	
				A177W +S1 A177F +S1	0,0232	1	
				A177W +S1 A177L	0,00681	1	
				A177W +S1 A177L +S1	0,85576	0	
				A177W +S1 A177W	0,03695	1	
Figure	Test	p value	statistics	post-hoc (in case of ANOVA) p<0,05	probability	significant	
S9H	Welch-ANOVA	9,17E-10	F(7, 24.22405)=26.19366	O1+S1 O1	2,95E-05	1	Patch clamp
				Orai1 F257A O1	0,27216	0	
				Orai1 F257A O1+S1	3,26E-05	1	
				Orai1 F257A + S1 O1	0,09335	0	
				Orai1 F257A + S1 O1+S1	1,77E-05	1	
				Orai1 F257A + S1 Orai1 F257A	0,20427	0	
				**Orai1 F257V O1**	0,00269	1	
				Orai1 F257V O1+S1	3,93E-05	1	
				Orai1 F257V Orai1 F257A	0,84994	0	
				Orai1 F257V Orai1 F257A + S1	0,30581	0	
				Orai1 F257V + S1 O1	0,14251	0	
				Orai1 F257V + S1 O1+S1	1,67E-05	1	
				Orai1 F257V + S1 Orai1 F257A	0,29897	0	
				Orai1 F257V + S1 Orai1 F257A + S1	0,99999	0	
				Orai1 F257V + S1 Orai1 F257V	0,43167	0	
				**Orai1 F257W O1**	0,0092	1	
				Orai1 F257W O1+S1	4,05E-05	1	
				Orai1 F257W Orai1 F257A	0,39217	0	
				Orai1 F257W Orai1 F257A + S1	0,45322	0	
				Orai1 F257W Orai1 F257V	0,89905	0	
				Orai1 F257W Orai1 F257V + S1	0,60837	0	
				Orai1 F257W + S1 O1	0,04428	1	
				Orai1 F257W + S1 O1+S1	0,00572	1	
				Orai1 F257W + S1 Orai1 F257A	0,06002	0	
				Orai1 F257W + S1 Orai1 F257A + S1	0,32673	0	

Figure	Test	p value	statistics	post-hoc (in case of ANOVA) p<0,05	probability	significant	
				Orai1 F257W + S1 Orai1 F257V	0,07213	0	
				Orai1 F257W + S1 Orai1 F257V + S1	0,26653	0	
				Orai1 F257W + S1 Orai1 F257W	0,08585	0	
Figure	Test	p value	statistics	post-hoc (in case of ANOVA) p<0,05	probability	significant	
S9I	OneWay ANOVA	0,30273	F(2,20)=1,26923	V102A A177F V102A	0,9508	0	Patch clamp
				V102A A177W V102A	0,17559	0	
				V102A A177W V102A A177F	0,18196	0	
Figure	Test	p value	statistics	post-hoc (in case of ANOVA) p<0,05	probability	significant	
S9J	Welch-ANOVA	0,00134	F(2, 15,65624)=10,29097	H134A A177F H134A	0,00154	1	Patch clamp
				H134A A177W H134A	0,00135	1	
				H134A A177W H134A A177F	0,93674	0	
Figure	Test	p value	statistics	post-hoc (in case of ANOVA) p<0,05	probability	significant	
S9K	Mann-Whitney test	NS(0,4501)					Patch clamp
Figure	Test	p value	statistics	post-hoc (in case of ANOVA) p<0,05	probability	significant	
S9L	OneWay ANOVA	0,24634	F(2,20)=1,50395	V102A F257A V102A	0,5652	0	Patch clamp
				V102A F257W V102A	0,23764	0	
				V102A F257W V102A F257A	0,1113	0	
Figure	Test	p value	statistics	post-hoc (in case of ANOVA) p<0,05	probability	significant	
S9M	Welch-ANOVA	6,87E-04	F(2, 14,33159)=12,81235	H134A F257A H134A	0,34616	0	Patch clamp
				H134A F257W H134A	0,00664	1	
				H134A F257W H134A F257A	0,00565	1	
Figure	Test	p value	statistics	post-hoc (in case of ANOVA) p<0,05	probability	significant	
S9N	Mann-Whitney test	S(0,00431)					Patch clamp
Figure	Test	p value	statistics	post-hoc (in case of ANOVA) p<0,05	probability	significant	
S10C	Welch-ANOVA	8,17E-09	F(5, 16.81257)=39.98828	O1 V181K + S1 O1 V181K	0,65341	0	Patch clamp
				**O1 T180F V181K O1 V181K**	1,42E-05	1	
				O1 T180F V181K O1 V181K + S1	0,00237	1	
				O1 T180F V181K + S1 O1 V181K	2,04E-05	1	
				O1 T180F V181K + S1 O1 V181K + S1	0,0027	1	
				O1 T180F V181K + S1 O1 T180F V181K	0,08192	0	
				**O1 T180W V181K O1 V181K**	1,47E-05	1	
				O1 T180W V181K O1 V181K + S1	0,00243	1	
				O1 T180W V181K O1 T180F V181K	0,99998	0	
				O1 T180W V181K O1 T180F V181K + S1	0,01896	1	
				O1 T180W V181K + S1 O1 V181K	1,61E-05	1	
				O1 T180W V181K + S1 O1 V181K + S1	0,00243	1	
				O1 T180W V181K + S1 O1 T180F V181K	0,695	0	

				O1 T180W V181K + S1 O1 T180F V181K + S1	0,94267	0	
				O1 T180W V181K + S1 O1 T180W V181K	0,53948	0	
Figure	Test	p value	statistics	post-hoc (in case of ANOVA) p<0,05	probability	significant	
S10D	Welch-ANOVA	2,25E-12	F(5,18.15727)=97.04707	O1+S1 O1	6,29E-07	1	Patch clamp
				T180F O1	0,97462	0	
				T180F O1+S1	3,43E-07	1	
				T180F + S1 O1	0,01654	1	
				T180F + S1 O1+S1	5,90E-08	1	
				T180F + S1 T180F	0,01866	1	
				T180W O1	0,59534	0	
				T180W O1+S1	3,36E-07	1	
				T180W T180F	0,57122	0	
				T180W T180F + S1	0,0113	1	
				T180W + S1 O1	0,99422	0	
				T180W + S1 O1+S1	2,42E-07	1	
				T180W + S1 T180F	1	0	
				T180W + S1 T180F + S1	0,01796	1	
				T180W + S1 T180W	0,75669	0	
